# Imparting amphiphobicity on single-crystalline porous materials

**DOI:** 10.1038/ncomms13300

**Published:** 2016-10-31

**Authors:** Qi Sun, Hongming He, Wen-Yang Gao, Briana Aguila, Lukasz Wojtas, Zhifeng Dai, Jixue Li, Yu-Sheng Chen, Feng-Shou Xiao, Shengqian Ma

**Affiliations:** 1 Key Lab of Applied Chemistry of Zhejiang Province and Department of Chemistry, Zhejiang University, Hangzhou 310028, China; 2 Department of Chemistry, University of South Florida, 4202 East Fowler Avenue, Tampa, Florida 33620, USA; 3 Electron Microscopy Centre, Zhejiang University, Hangzhou 310027, China; 4 ChemMatCARS, Center for Advanced Radiation Sources, The University of Chicago, 9700 South Cass Avenue, Argonne, Illinois 60439, USA

## Abstract

The sophisticated control of surface wettability for target-specific applications has attracted widespread interest for use in a plethora of applications. Despite the recent advances in modification of non-porous materials, surface wettability control of porous materials, particularly single crystalline, remains undeveloped. Here we contribute a general method to impart amphiphobicity on single-crystalline porous materials as demonstrated by chemically coating the exterior of metal-organic framework (MOF) crystals with an amphiphobic surface. As amphiphobic porous materials, the resultant MOF crystals exhibit both superhydrophobicity and oleophobicity in addition to retaining high crystallinity and intact porosity. The chemical shielding effect resulting from the amphiphobicity of the MOFs is illustrated by their performances in water/organic vapour adsorption, as well as long-term ultrastability under highly humidified CO_2_ environments and exceptional chemical stability in acid/base aqueous solutions. Our work thereby pioneers a perspective to protect crystalline porous materials under various chemical environments for numerous applications.

## Abstract (web summary)

The inherent instabilities of metal-organic frameworks (MOFs) in the presence of water or organic compounds have limited their real-world applicability. Here, Ma and co-workers present a coating strategy to fabricate MOFs with amphiphobic surfaces, simultaneously protecting them from moisture and organic vapours.

The custom design of surfaces with controlled wettability properties has been attracting continuous attention from both academia and industry^1^,^2^,^3^,^4^,^5^,^6^,^7^. In particular, amphiphobic surfaces^8^,^9^,^10^,^11^,^12^ that repel water and compounds with low surface tension (for example, oils) are of great interest due to their various prospective applications, such as oil transportation^13^, microfluidics^14^ and nano-object manipulation^15^. Albeit tremendous efforts have been devoted to creating such amphiphobic surfaces on a variety of substrates over the past several years^8^,^9^,^10^,^11^,^12^, the impartment of amphiphobicity on porous materials, specifically single crystalline, has not yet been explored.

As an emerging class of porous materials with high single crystallinity, metal-organic frameworks (MOFs)^16^,^17^,^18^, which feature amenability to design, high surface areas, tunable pore sizes and tailorable functionality, have recently been extensively investigated for applications in gas storage^19^,^20^, separation^21^,^22^, carbon capture^23^,^24^, catalysis^25^,^26^,^27^,^28^, sensing^29^,^30^ and so on (refs 31, 32, 33, 34). However, an issue for their wide applications in practice includes the performance under a variety of environments (for example, stability in humid conditions, interferences by organic vapours in the atmosphere^35^,^36^,^37^,^38^), which necessitate the sophisticated control of the surface wettability of MOFs. Recent studies indicate the observation of framework degradation on the exposure of MOFs to wet CO_2_, even for those claimed with moisture stability^39^,^40^. To repel water molecules, thereby protecting MOFs against hydrolysis^41^,^42^,^43^,^44^,^45^,^46^,^47^,^48^,^49^,^50^,^51^,^52^,^53^ while preserving crystallinity and intact porosity, some approaches have been developed to impart hydrophobicity/superhydrophobicity on the exterior surfaces of MOFs. For example, Jiang and colleagues^54^ recently developed a facile yet general coating approach to modify hydrophobic polydimethysiloxane on the surfaces of MOF materials, which exhibited significant enhancement in moisture/water stability. However, some environments coexist with water and organic compounds. Therefore, amphiphobicity is needed to repel both water and organic molecules, to prevent the water attack while minimizing the interferences by organic compounds. Nonetheless, amphiphobic MOFs have not yet been achieved, despite their intriguing properties and attractive potentials for a variety of applications^13^,^14^,^15^.

To impart amphiphobicity on the MOFs, in this contribution we rationally designed vinyl-functionalized linkers for targeted construction of MOFs, where the vinyl groups can remain intact during the formation process of MOF crystals, yet are sufficiently reactive for further chemical modifications^55^. After controllable functionalization of the exterior amphiphilic crystal surfaces with perfluoroalkyl groups via thiol-ene reaction, the resultant MOFs exhibit both superhydrophobicity and oleophobicity, while retaining high crystallinity and intact porosity.

## Results

### Synthesis of vinyl prefunctionalized MOF

As a representative example of MOFs, a vinyl-functionalized crystalline zeolite imidazole framework was prepared using the protocol reported in the literature^56^ and it is isostructural with ZIF-8 as revealed by single-crystal X-ray diffraction (XRD) analysis (Supplementary Fig. 1). The guest solvent-free crystal product is designated as ZIF-8-V with a formula of Zn(C_5_H_5_N_2_)_2_ and the retention of vinyl groups on the linkers during the crystal formation process was further confirmed by liquid ^1^H NMR analysis of the digested ZIF-8-V sample (Supplementary Figs 2 and 3).

### Crystal surface coating with perfluoroalkyl groups

**Figure 1:**
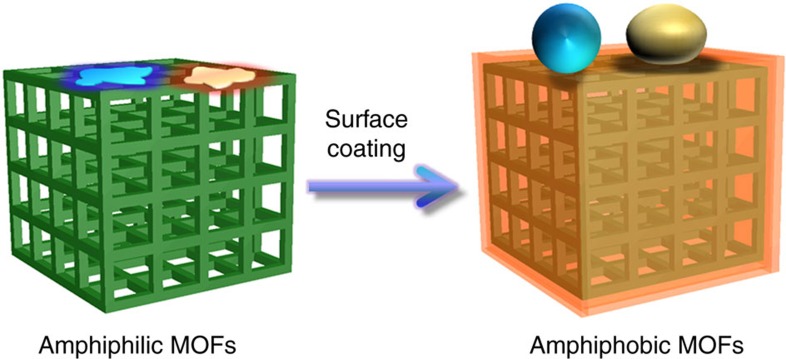
Amphiphobic surface engineering for MOFs. The resultant MOFs exhibit both superhydrophobicity and oleophobicity, while retaining high crystallinity and intact porosity.

**Figure 2:**
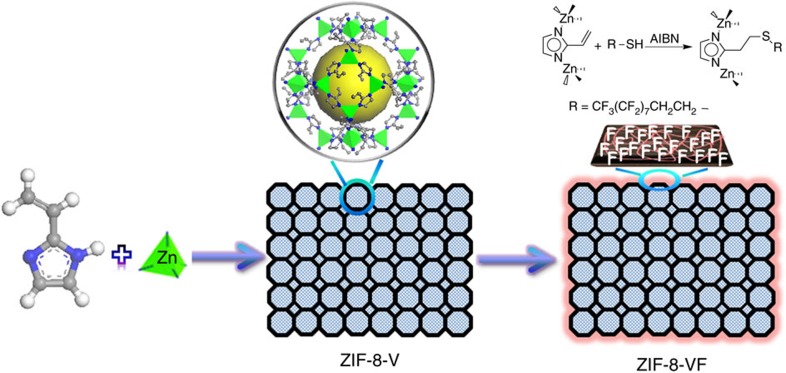
Schematic illustration to impart amphiphobicity on ZIF-8-V. Synthetic route to create amphiphobic surface via grafting perfluoroalkyl groups on the exterior surface of the ZIF-8-V crystal.

To controllably introduce the fluorinated groups onto the exterior surface of ZIF-8-V, we hypothesize that, if a relatively bulky fluorinated compound that cannot permeate into the pore of the material is employed, the reaction would only occur on the exterior surface of the crystals. In addition, if these fluorocarbon chains are long enough, the surface energy of the sample would be significantly reduced^9^,^10^ (Fig. 1). To demonstrate this proof-of-concept, 1H,1H,2H,2H-perfluorodecanethiol was chosen as a typical compound for the surface modification of ZIF-8-V crystals, which was achieved via the thiol-ene click reaction^57^ to afford ZIF-8-VF (Fig. 2).

### Structural characterization

**Figure 3:**
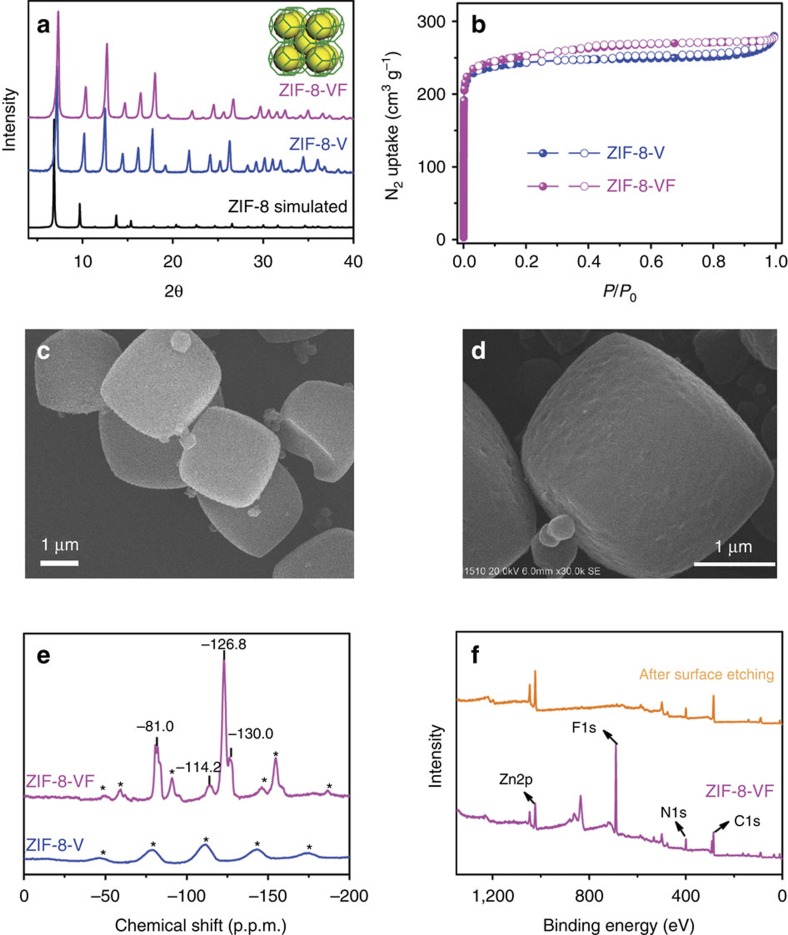
Structural characterization. (**a**) PXRD patterns, (**b**) N_2_ sorption isotherms measured at 77 K, (**c**) SEM image of ZIF-8-V, (**d**) SEM image of ZIF-8-VF, (**e**) ^19^F MAS NMR curves and (**f**) X-ray photoemission spectra of ZIF-8-VF before and after surface Ar^+^ ions etching (*side band).

Figure 3a shows powder XRD (PXRD) patterns of ZIF-8-V and ZIF-8-VF, which agree well with the calculated ones of ZIF-8-V, indicating the good retention of crystallinity and structural integrity during the post-synthetic modification process. N_2_ sorption isotherms collected at 77 K (Fig. 3b) reveal that both ZIF-8-V and ZIF-8-VF exhibit the classic type I adsorption behaviour, a characteristic of microporous materials. Derived from the N_2_ adsorption data, ZIF-8-V and ZIF-8-VF have similar Brunauer-Emmett-Teller (BET) surface areas (816 and 850 m^2^ g^−1^) and pore volumes (0.42 cm^3^ g^−1^), suggesting that the postmodification process should primarily occur on the exterior surface of the crystals with negligible blockage of the pores in the pristine material, thereby still accessible for guest gas molecules. The scanning electron microscopy (SEM) images (Fig. 3c,d) show that there is little change in the overall morphology of the crystals after the chemical modification reaction but the surfaces of the ZIF-8-VF crystals are rougher than those of ZIF-8-V crystals, further indicating the occurrence of the reaction on the exterior surface of the crystals. It is worth noting that such roughness of the surface has been reported to be beneficial to the enhancement of the hydrophobicity and oleophobicity^8^.

To identify the surface coating, the ^13^C and ^19^F MAS NMR spectra of ZIF-8-V and ZIF-8-VF were collected (Fig. 3e and Supplementary Fig. 4). Albeit no distinct difference in the ^13^C NMR spectra are observed for the two samples, ZIF-8-VF shows clear ^19^F NMR signals with the same chemical shifts as those of the 1H,1H,2H,2H-perfluorodecanethiol compound (Supplementary Fig. 5), which can be attributed to the much higher natural abundance of ^19^F nuclei than that of ^13^C nuclei. In addition, the Fourier transform infrared spectra of the ZIF-8-VF show the characteristic bands of C-F at 1,241 and 1,211 cm^−1^ as compared with the pristine ZIF-8-V (Supplementary Fig. 6). These results indicate that the perfluoroalkyl groups have been successfully introduced on ZIF-8-V, but the grafting degree is relatively low. To quantify the degree of postsynthetic modification, the ZIF-8-VF crystals were digested and analysed by the liquid ^1^H NMR spectroscopy (Supplementary Fig. 7). The results show that 98% of vinyl groups are still intact, which means that ∼2% of the vinyl groups are involved in the thiol-ene reaction probably occurring only on the exterior surface of the ZIF-8-V. To further prove the exterior surface modification, X-ray photoemission spectroscopy experiments were conducted (Fig. 3f and Supplementary Fig. 8), which reveal that ZIF-8-VF exhibits very strong signals associated with the F species. However, after the surface removal by Ar^+^ ion-etching treatment (2 KeV, 100 s), the amount of residual F species is very small. These results suggest that the perfluoroalkyl groups should be mainly attached to the vinyl groups on the exterior surface of ZIF-8-V crystals, which is further confirmed by the energy-dispersive X-ray mapping of the ZIF-8-VF sample before and after surface plasma cleaning process (Supplementary Figs 9 and 10).

### Examination of amphiphobic properties

**Figure 4:**
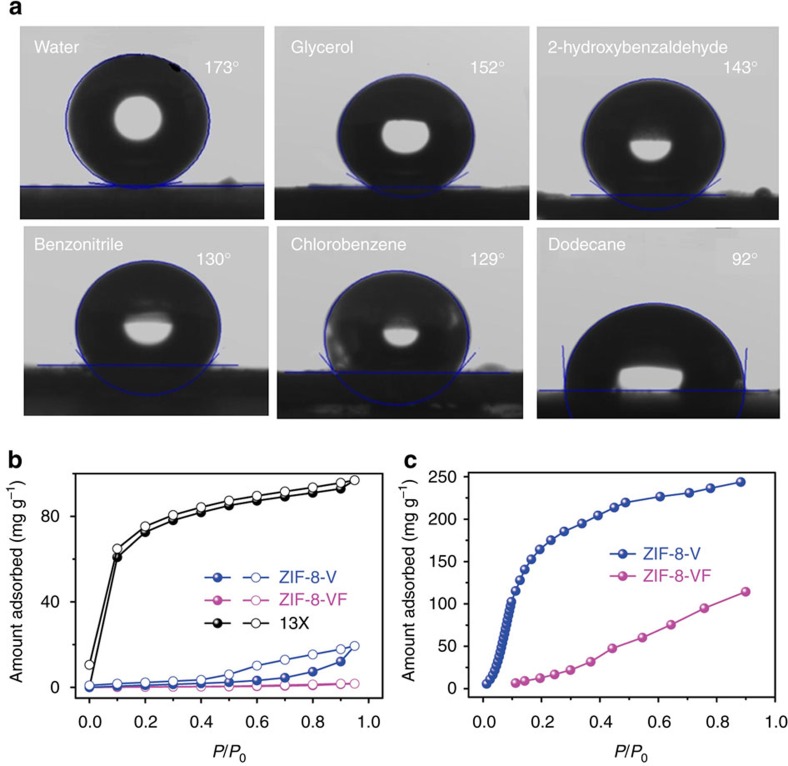
Wettability tests and vapour sorption performance. (**a**) Contact angles of various liquid on the pressed pellet made using ZIF-8-VF sample, (**b**) water adsorption (solid symbols) and desorption (open symbols) isotherms collected at 298 K and (**c**) toluene adsorption isotherms collected at 298 K.

The surface wettability of ZIF-8-V and ZIF-8-VF was characterized by contact-angle measurements. Figure 4a shows contact angles of water and a series of organic compounds on the surface of ZIF-8-VF. Notably, the contact angle of water on ZIF-8-VF sample is as high as 173°, indicating its extraordinarily superhydrophobic feature. In contrast, ZIF-8-V gives the water contact angle at 89° (Supplementary Fig. 11). These results confirm that the surface coating of perfluoroalkyl groups significantly enhances the hydrophobicity of the material. Furthermore, when a series of organic compounds with different surface tensions, including glycerol, 2-hydroxybenzaldehyde, benzonitrile, chlorobenzene and dodecane, were contacted with the surface of ZIF-8-VF, the contact angles at 150°, 143°, 130°, 129° and 92°, respectively, were observed, indicating the oleophobic feature of ZIF-8-V. The results of the contact-angle experiments coupled with the permanent porosity, as revealed from the N_2_ sorption measurement, thereby identify ZIF-8-VF as an amphiphobic porous material. In contrast, the contact angles of 2-hydroxybenzaldehyde, benzonitrile, chlorobenzene and dodecane on ZIF-8-V are <5° (Supplementary Fig. 12), indicating its superoleophilic nature. The superhydrophobic behaviour of the ZIF-8-VF was further illustrated by water vapour adsorption experiments (Fig. 4b). As a comparison, hydrophilic zeolite 13 × exhibits strong affinity for water even at very low relative humidity (*P/P*_0_<0.1). Hydrophobic ZIF-8-V exhibits a hysteresis loop at relative humidity >0.5, indicating that ZIF-8-V is capable of adsorbing water at high humidity. Interestingly, ZIF-8-VF adsorbs a negligible amount of water even at *P/P*_0_ up to 0.9, which is anticipated to be effective to preclude the entrance of water within the interior pores of the MOF^58^,^59^,^60^. Toluene sorption isotherms collected at 298 K also reveal quite different adsorption behaviours for ZIF-8-V and ZIF-8-VF (Fig. 4c). For example, when *P/P*_0_ is 0.15, it is observed that the adsorption capacity of toluene in ZIF-8-V and ZIF-8-VF is 143 and 7 mg g^−1^, respectively. The repellency of ZIF-8-VF for toluene should stem from its excellent oleophobicity. The above results highlight that the surface-coated amphiphobic perfluoroalkyl groups serve as a shield to effectively prevent the access of water and organic compounds into the micropores of MOFs.

### Investigation of chemical shielding effect

**Figure 5:**
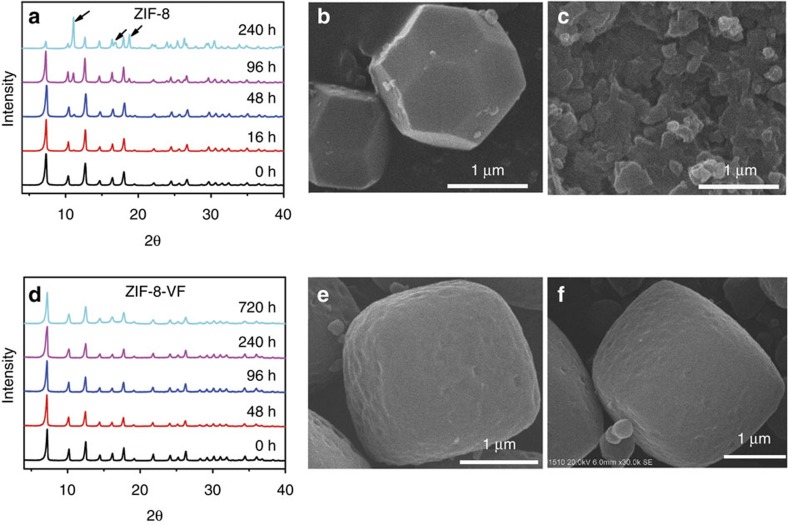
Stability comparison of ZIF-8 and ZIF-8-VF. (**a**,**d**) Selected PXRD patterns of ZIF-8 and ZIF-8-VF ageing under 1 atm of water saturated CO_2_ at 45 °C for different duration times. (**b**,**c**) SEM images of ZIF-8 before and after ageing under the above conditions for 240 h. (**e**,**f**) SEM images of ZIF-8-VF before and after aging under the above conditions for 720 h. Main peaks of unknown crystalline phase are marked with black arrows.

The long-term stability under practical environments (for example, humidity) has been recognized as an issue for MOFs, which can be accessed via the method of accelerated ageing^39^. To evaluate the chemical shielding effect resulting from amphiphobicity, the PXRD patterns of various ZIF materials that are exposed to 100% relative humidity under CO_2_ atmosphere at 45 °C were monitored for different duration times. When ZIF-8, one of the claimed very stable MOF materials^61^,^62^, was aged under the above conditions for 16 h, it was observed that some additional peaks associated to an unknown phase appeared, as shown by the black arrows in Fig. 5a. Moreover, the ratio of the unknown phase increases with the increase of exposure time. For instance, after 240 h, a large portion of ZIF-8 was transformed, indicative of structural degradation of ZIF-8. This conclusion is supported by N_2_ sorption studies at 77 K, suggesting a remarkable reduction in the surface area (from 957 to 378 m^2^ g^−1^; Supplementary Fig. 13), as well as SEM images showing clear cracks of the crystals (Fig. 5b,c). Similar changes in the PXRD patterns were also observed for ZIF-8-V (Supplementary Fig. 14). In striking contrast, ZIF-8-VF does not experience any change in the PXRD patterns, even after ageing under the above conditions over 720 h. The SEM images show that the ZIF-8-VF sample has maintained perfect crystal morphology and its surface area is also fully retained (802 m^2^ g^−1^; Supplementary Fig. 15). These results highlight that the amphiphobic surface can serve as a chemical shield to effectively prevent ZIF-8-VF from being attacked by the mixture of H_2_O and CO_2_.

## Discussion

**Figure 6:**
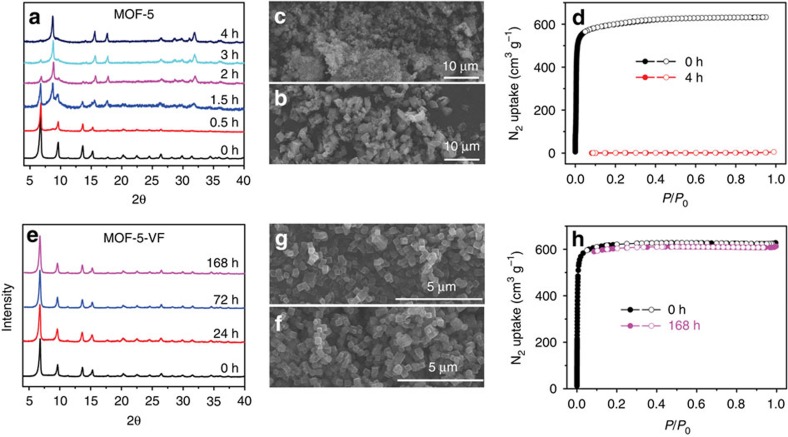
Stability comparison of MOF-5 and MOF-5-VF. (**a**,**e**) Selected PXRD patterns of MOF-5 and MOF-5-VF ageing under 1 atm of water saturated CO_2_ at 45 °C for different duration times. (**b**,**c**) SEM images and (**d**) N_2_ sorption isotherms of MOF-5 before and after ageing under the above conditions for 4 h. (**f**,**g**) SEM images and (**h**) N_2_ sorption isotherms of MOF-5-VF before and after aging under the above conditions for 168 h.

To demonstrate the general applicability of the strategy presented herein, vinyl-functionalized MOF (MOF-5-V; Supplementary Fig. 16) isostructural with MOF-5, which is notoriously water/moisture unstable, was synthesized using the custom-designed ligand of 2-vinylterephthalic acid (Supplementary Fig. 17). After surface chemical coating of the perfluoroalkyl groups (Supplementary Figs 18–22), the resultant material (MOF-5-VF) demonstrates amphiphobic property (Supplementary Figs 23 and 24), which renders it with extraordinary tolerance to the humidified CO_2_, as evidenced by its well-retained crystallinity, morphology and surface area after ageing under the aforementioned conditions for 7 days (Fig. 6e–h). In sharp contrast, as observed from PXRD patterns, MOF-5 starts to transform to the non-porous MOF-69 under the humidified CO_2_ environment within <1.5 h and such a transformation almost finishes after 4 h as indicated by the complete disappearance of MOF-5 phase in the PXRD patterns. Correspondingly, the SEM images show that the MOF-5 crystals underwent serious corrosion along with the complete loss of its porosity after 4 h (Fig. 6a–d). These results highlight the chemical shielding effect of amphiphobicity in protecting the highly unstable MOF-5 analogues under various conditions such as humidified CO_2_ atmosphere, environment with high humidity and aqueous solutions (Supplementary Figs 25–27). The greatly enhanced stability of amphiphobic MOFs over their pristine ones in both acidic and basic aqueous solutions further underscore the chemical shielding effect of amphiphobicity (Supplementary Figs 28–37).

In summary, we have demonstrated the successful impartment of amphiphobicity (that is, superhydrophobic and oleophobic) on the exterior surface of the highly single-crystalline porous materials of MOFs. Such amphiphobic surface can serve as a chemical shield to effectively prevent the MOFs from being attacked by water and organic compounds, thereby bestowing the MOFs with ultrastability towards moisture/water and humidified CO_2_. Our approach contributed herein to create amphiphobic surface has little impact on the crystallinity and porosity of the pristine MOF materials, thereby pioneering a perspective to protect crystalline porous materials under various chemical environments for numerous applications.

## Methods

### Materials and measurements

Commercially available reagents were purchased in high purity and used without purification. Solvents were purified according to standard laboratory methods. Tetrahydrofuran (THF) was distilled over LiAlH_4_. Dimethylformamide (DMF) was distilled over CaH_2_. Nitrogen sorption isotherms at the temperature of liquid nitrogen were measured using Micromeritics ASAP 2020M and Tristar System. The samples were outgassed for 12 h at 100 °C before the measurements. ^1^H NMR spectra were recorded on a Bruker Avance-400 (400 MHz) spectrometer. Chemical shifts are expressed in p.p.m. downfield from tetramethylsilane (TMS) at *δ*=0 p.p.m. and *J* values are given in Hz. SEM was performed on a Hitachi SU 1510. PXRD patterns were measured with a Rigaku Ultimate VI X-ray diffractometer (40 kV, 40 mA) using CuKα (*λ*=1.5406 Å) radiation. Photographs of water and organic compounds on the surface of the samples in the pressed pellet form were measured with SL200KB (USA KNO Industry, Co.), equipped with a charge-coupled device camera. X-ray photoemission spectra were performed on a Thermo ESCALAB 250 with Al Kα irradiation at *θ*=90° for X-ray sources and the binding energies were calibrated using the C1s peak at 284.9 eV. An Ar^+^ sputter beam (2 keV, 100 s) was used for depth profiling of ZIF-8-VF and MOF-5-VF after the initial data was collected. High-angle annular dark field scanning, scanning transmission electron microscopic imaging and energy-dispersive X-ray spectroscopy mapping were carried out by Titan ChemiSTEM operated at 200 kV. Water adsorption and desorption isotherms were obtained via SMS Instruments DVS Advantage. The balance has a sensitivity of 0.1 μg. These isotherms were measured at 25 °C by monitoring the weight change of the sample as a function of relative humidity of water. The relative humidity of water was stepped up from 0 to 98% with an increment of 10% in each step and then was stepped down to 0%. Real-time weight, temperature and relative humidity were recorded. Toluene adsorption isotherms were measured via Micromeritics 3Flex. These isotherms were collected at 25 °C by monitoring the volume change.

### Synthesis of single-crystal ZIF-8-V

Single crystal of ZIF-8-V was obtained by slowly evaporating the mixture of trimethylamine (3 μl) and cyclohexane into a DMF (2 ml) solution of Zn(NO_3_)_2_·6H_2_O (0.033 mmol) and 2-vinyl-imidazole (0.1 mmol) for 7 days at room temperature.

### Synthesis of powder ZIF-8-V crystal

2-Vinyl-imidazole (0.094 g, 1.0 mmol) and zinc nitrate hexahydrate [Zn(NO_3_)_2_·6H_2_O] (0.149 g, 0.50 mmol) in DMF (15 ml) was placed in a desiccator under an atmosphere of the mixture of triethylamine (5 ml) and cyclohexane (200 ml). The reaction was allowed to proceed at room temperature for 48 h. The crystalline powder was obtained by centrifugation, washed with methanol (3 × 25 ml) and activated with methanol (3 × 25 ml) for 3 days before being dried under vacuum at room temperature. Yield (0.09 g, 72 %) CHN calculated for C_10_H_10_N_4_Zn: C, 48.0; H, 4.0; N, 22.3%. Found: C, 48.3; H, 4.40; N, 20.1%.

### Covalent post-synthetic modification of ZIF-8-V

Activated ZIF-8-V powder (0.10 g) was suspended in (trifluoromethyl)benzene (10 ml) solution containing 10 v/v % 1H,1H,2H,2H-perfluorodecanethiol and catalytic amount of azobisisobutyronitrile. The reaction was carried out at 60 °C for 10 h under N_2_, to attach perfluoroalkyl groups on the crystal surface by the thiol-ene click reaction. The product denoted as ZIF-8-VF was obtained by centrifugation, washed with methanol (3 × 25 ml) and dried under vacuum at room temperature. CHN found for ZIF-8-VF: C, 49.3; H, 4.49; N, 18.5%.

### Data availability

The authors declare that the data supporting the findings of this study are available within the article and its Supplementary Information files and from the corresponding author upon reasonable request.

## Additional information

**How to cite this article:** Sun, Q. *et al*. Imparting amphiphobicity on single-crystalline porous materials. *Nat. Commun.* **7,** 13300 doi: 10.1038/ncomms13300 (2016).

**Publisher's note:** Springer Nature remains neutral with regard to jurisdictional claims in published maps and institutional affiliations.

## Supplementary Material

Supplementary Information Supplementary Figures 1-37, Supplementary Tables 1-2, Supplementary Methods and Supplementary References
