# Supplementary material for: Imparting amphiphobicity on single-crystalline porous materials
Source: Nat Commun. 2016 Oct 31;7:13300. doi: 10.1038/ncomms13300 (PMC5095586; doi:10.1038/ncomms13300)
Supplement: Supplementary Information — Supplementary Figures 1-37, Supplementary Tables 1-2, Supplementary Methods and Supplementary References [file ncomms13300-s1.pdf]

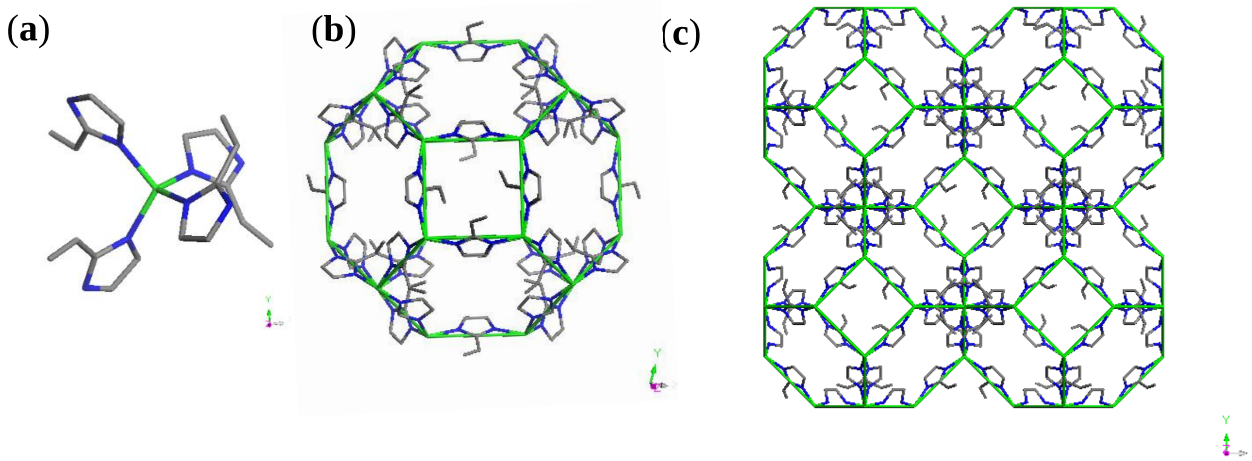

Supplementary Figure 1 | Representations of the crystal structure of ZIF-8-V.

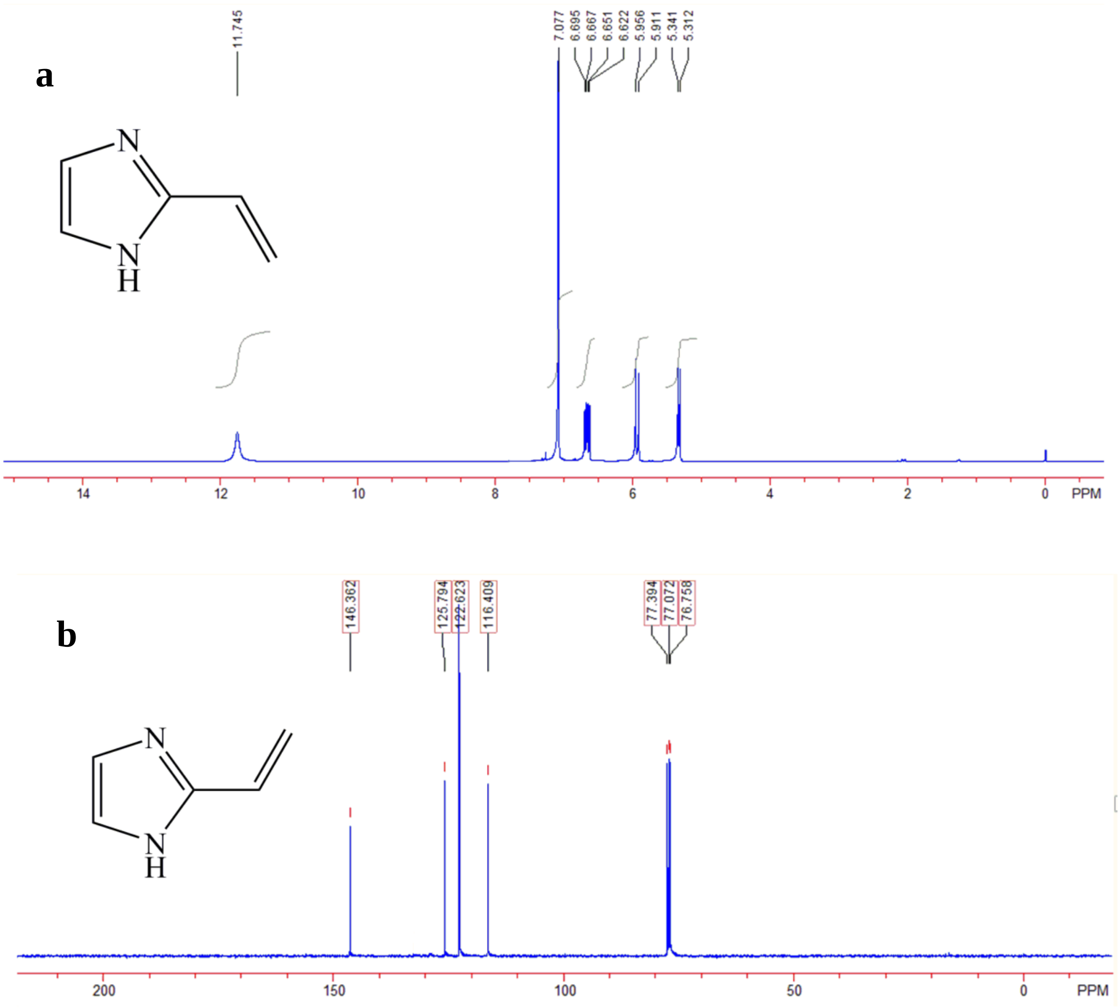

Supplementary Figure 2 | Liquid NMR spectra. (a) ^{1}H NMR, and (b) ^{13}C NMR spectra of 2-vinyl-imidazole.

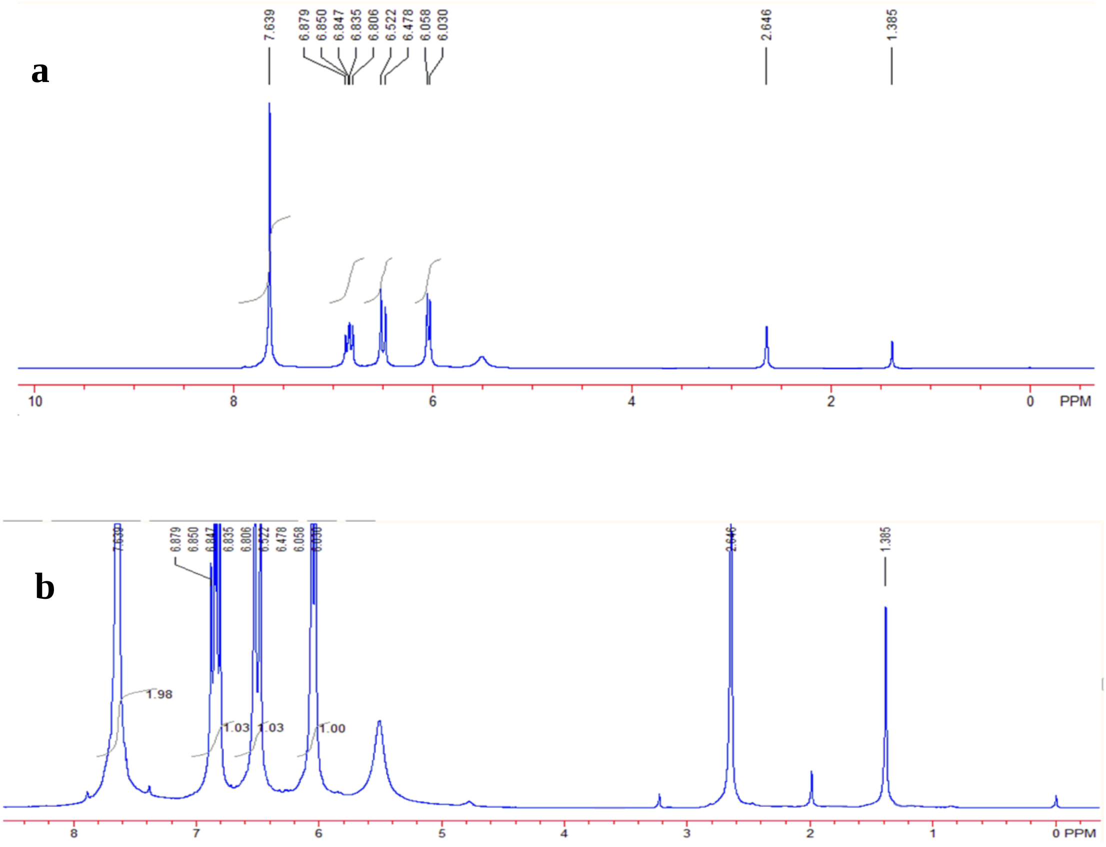

Supplementary Figure 3 | Liquid NMR spectra of digested ZIF-8-V sample (a) and corresponding enlarged spectrum (b). ^{1}H NMR spectrum of the digested sample displayed the same relative integrated peak intensities compared with that of the as-synthesized 2-vinyl-imidazole linkers, although, some chemical shift, stemming from the protonation of imidazole ring by DCl, was observed. These results indicate that 2-vinyl-imidazole linkers are stable under the synthetic conditions of ZIF-8-V.

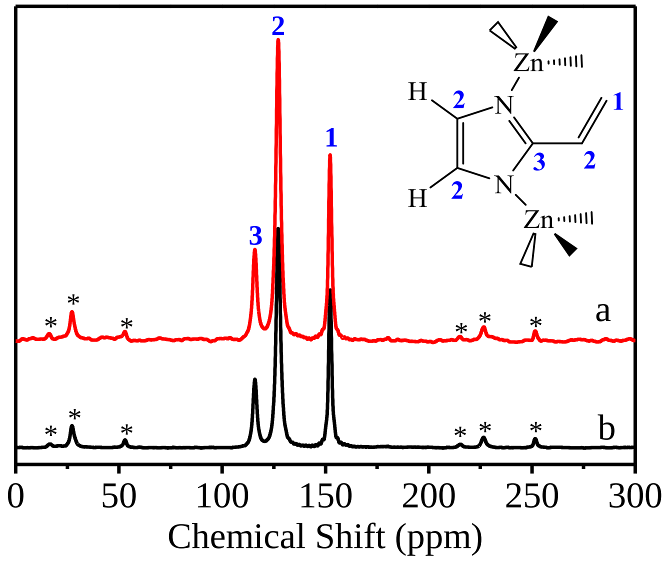

Supplementary Figure 4 | ^{13}C solid state NMR spectra of (a) ZIF-8-V and (b) ZIF-8-VF. There is almost no identifiable difference between the two samples, suggesting that the amount of incorporated perfluoroalkyl groups should be very small.

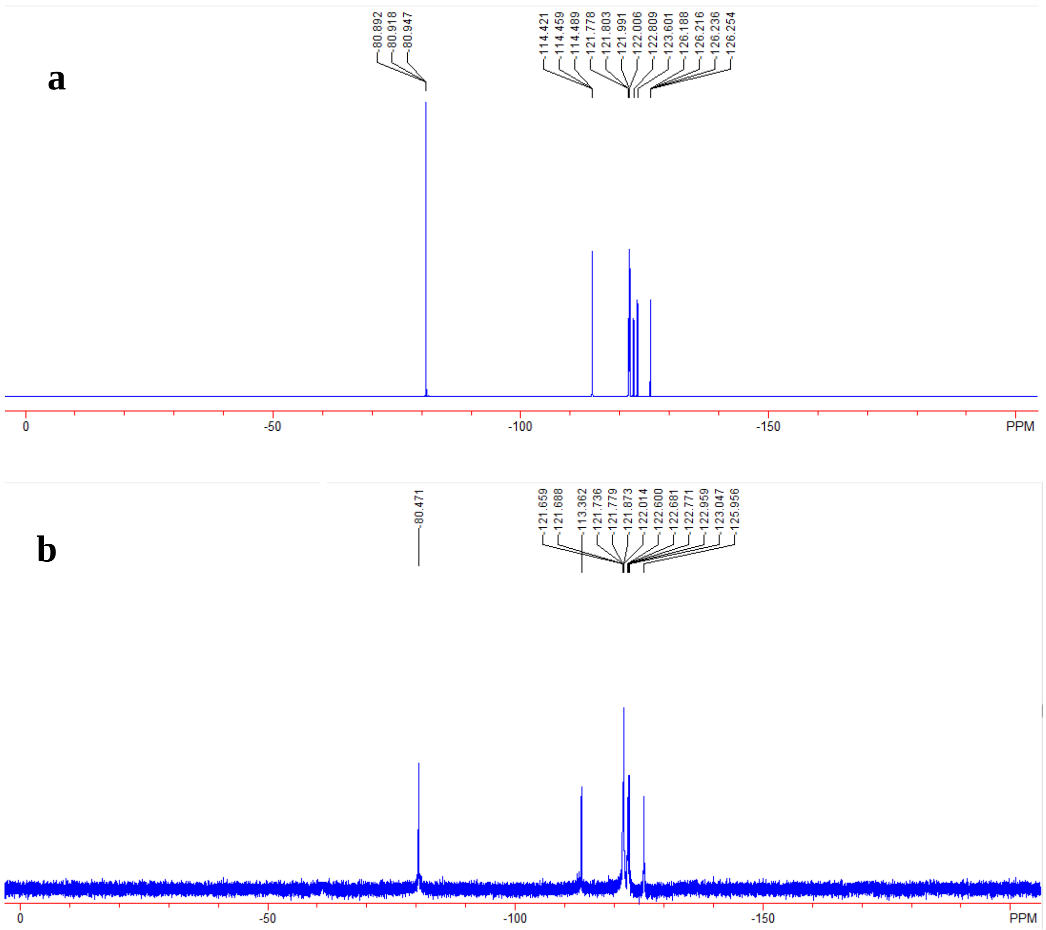

Supplementary Figure 5 | Liquid ^{19}F NMR spectra.

(**a**)1H,1H,2H,2H-perfluorodecanethiol compound, and (**b**) digested ZIF-8-VF sample. The chemical shift of F species in the digested ZIF-VF sample are similar to that of the 1H,1H,2H,2H-perfluorodecanethiol compound.

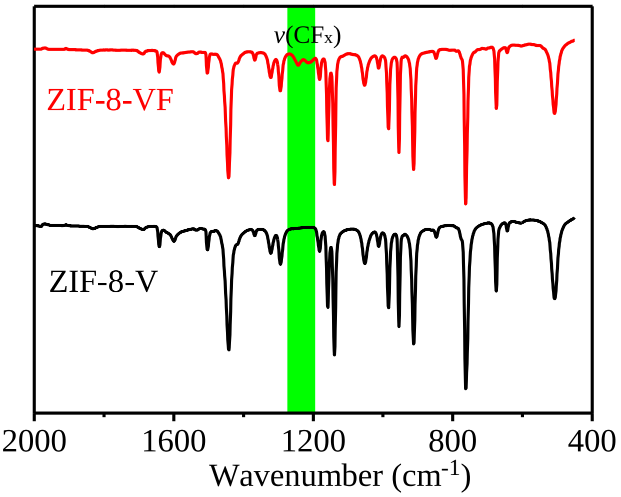

Supplementary Figure 6 | FT-IR spectra of ZIF-8-V and ZIF-8-VF. The appearance of characteristic bands of C-F at 1241 and 1211 cm^{-1} in ZIF-8-VF indicates the successful grafting of perfluoroalkyl groups.[1]

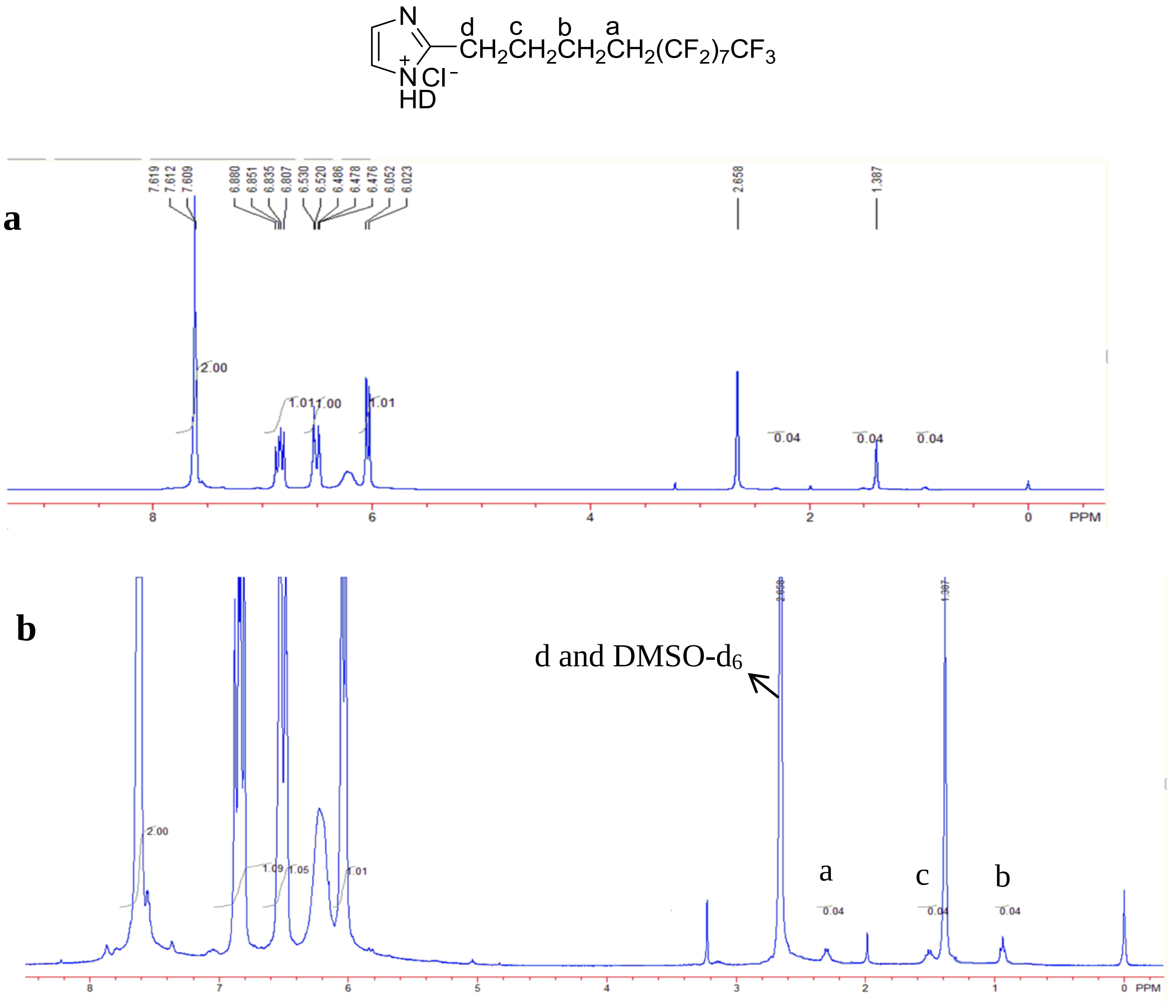

Supplementary Figure 7 | Liquid NMR spectra of digested ZIF-8-VF sample (a) and corresponding enlarged spectrum (b). The chemical shift of d is overlapped with d_6-DMSO. These spectra reveal that only about 2.0 % of vinyl groups have been grafted with perfluoroalkyl groups.

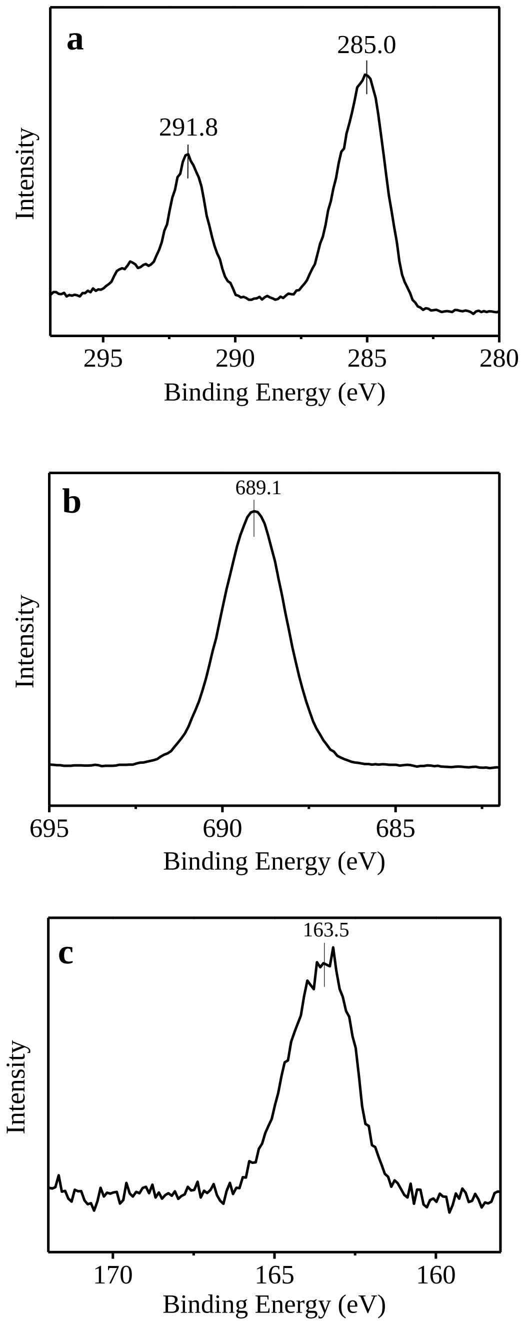

Supplementary Figure 8 | XPS spectra of ZIF-8-VF sample (a) C1s, (b) F1s, and (c) S2p. These strong XPS signals of C1s at relatively high binding energy, F1s, and S2p indicate that there are a lot of perfluoroalkyl groups grafted on the surface of the ZIF-8-VF sample.

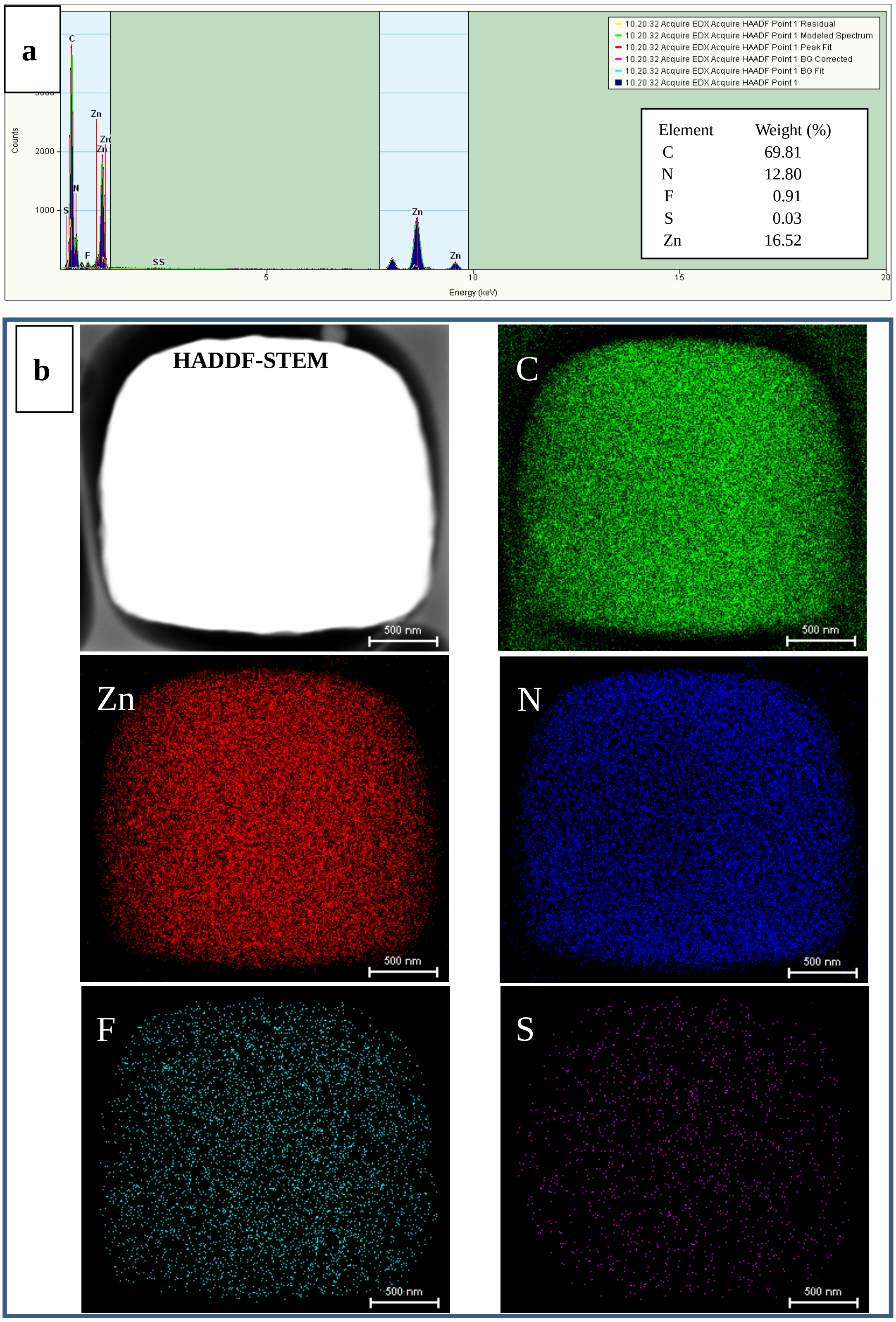

Supplementary Figure 9 | Energy-dispersive X-ray (EDX) spectroscopy results (a) and HADDF-STEM and corresponding element mapping (b) of ZIF-8-VF sample. Signals were accumulated for 3 min.

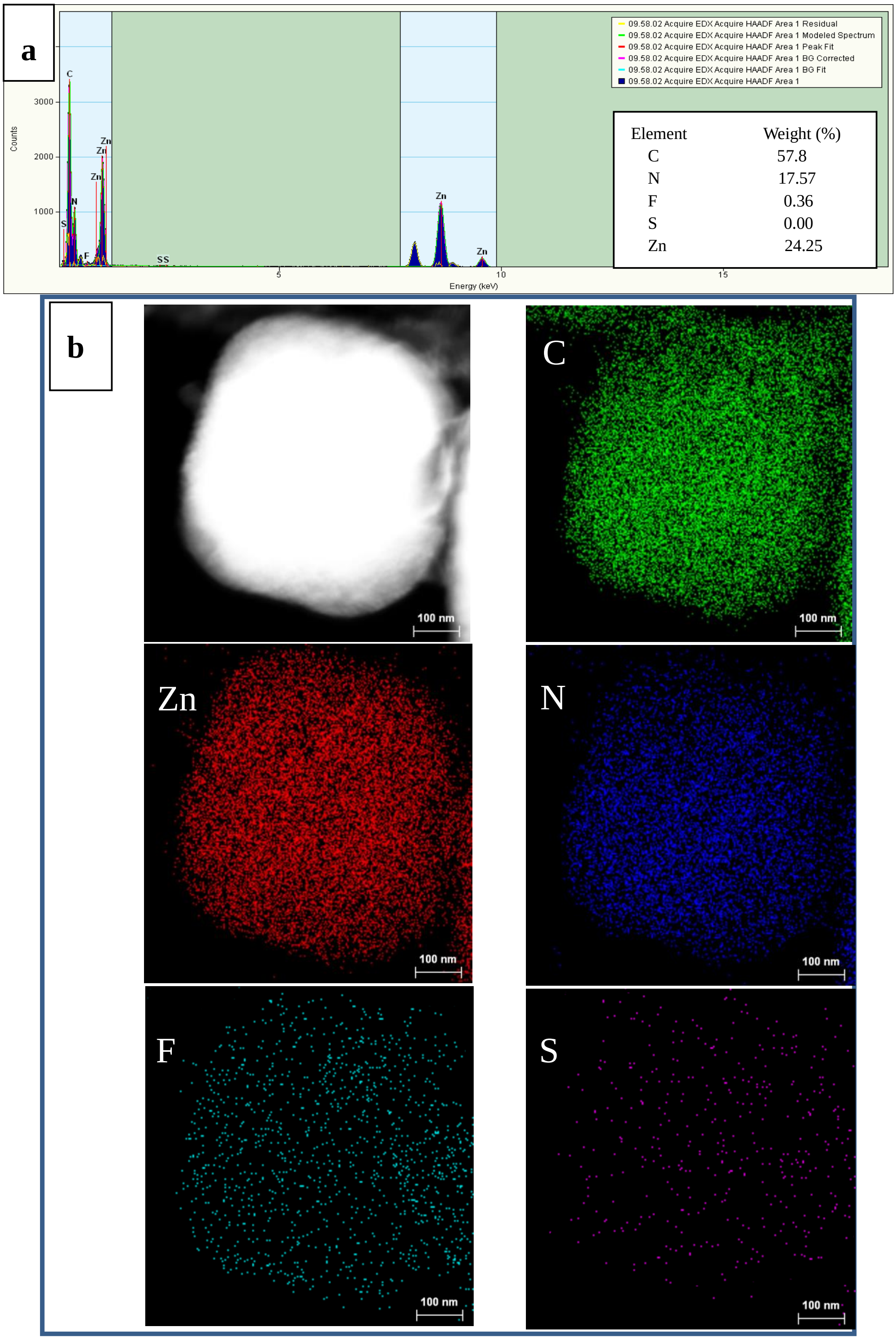

Supplementary Figure 10 | Energy-dispersive X-ray (EDX) spectroscopy results (a) and HADDF-STEM and corresponding element mapping (b) of ZIF-8-VF sample after surface cleaning by plasma (25% O_2 and 75% Ar) for 2 min. Signals were accumulated for 3 min.

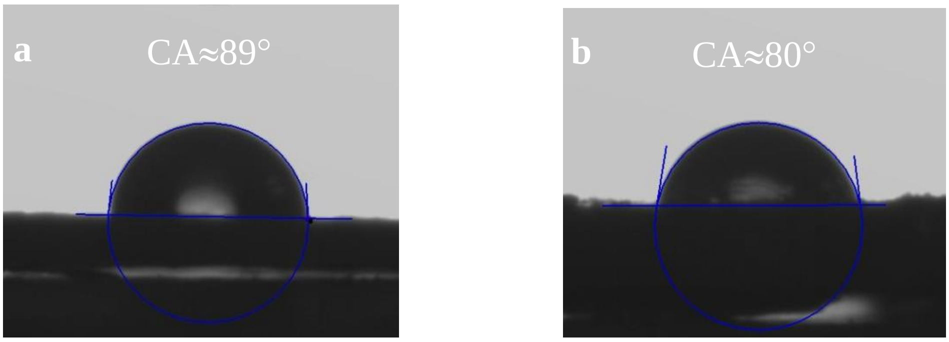

Supplementary Figure 11 | Contact angles of water on the pressed pellet of (a) ZIF-8-V and (b) ZIF-8.

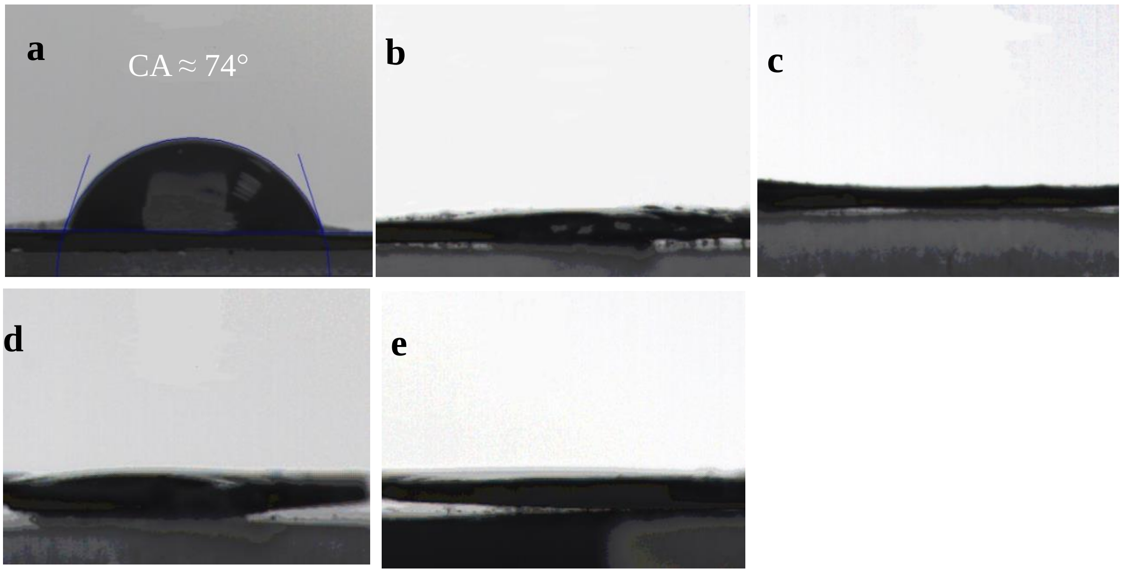

Supplementary Figure 12 | Contact angles of various organic compounds on the pressed pellet of ZIF-8-V. (**a**) glycerol, (**b**) 2-hydroxybenzaldehyde, (**c**) benzonitrile, (**d**) chlorobenzene, and (**e**) dodecane.

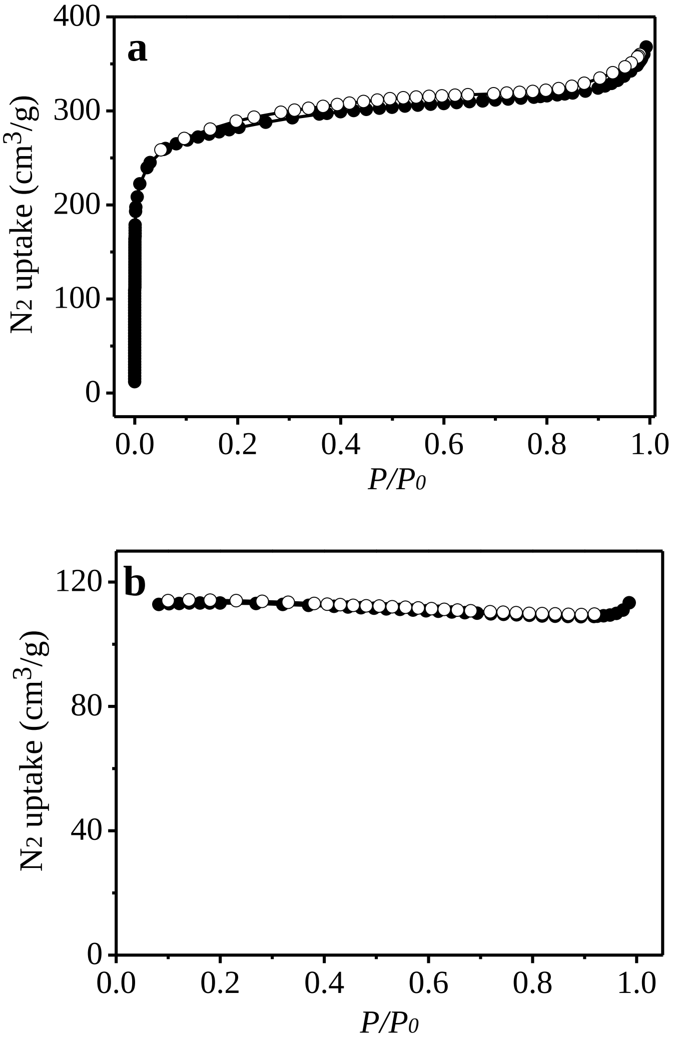

Supplementary Figure 13 | N_2 sorption isotherms. (**a**) activated ZIF-8, and (**b**) ZIF-8 after aging at 100% relative humidity and 45 °C under CO_2 for 10 d. The sharp decrease of the BET surface area (from 957 m^2/g to 378 m^2/g) indicates the structural decomposition of the ZIF-8 sample after long-term exposure to humidified CO_2.

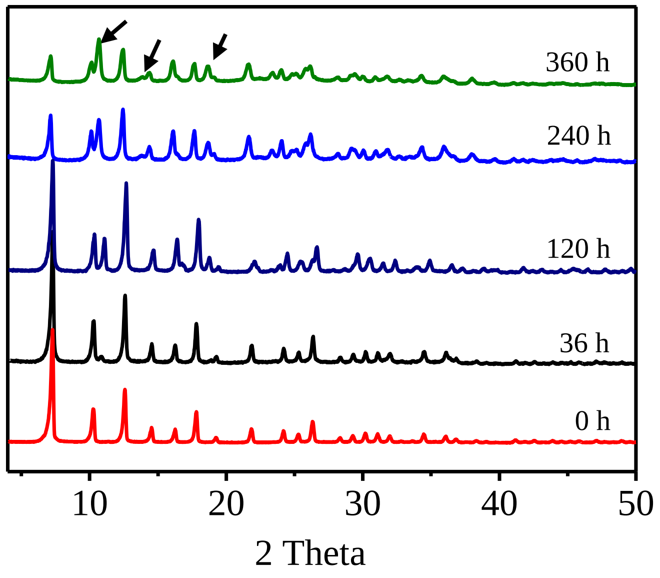

Supplementary Figure 14 | Selected PXRD patterns of ZIF-8-V aged at 100% relative humidity and 45 °C under CO_2 for different duration times. Main peaks of unknown crystalline phase are marked with black arrows. These results indicate the ZIF-8-V sample experienced a chemical decomposition for long-term exposure in humidified CO_2 environment.

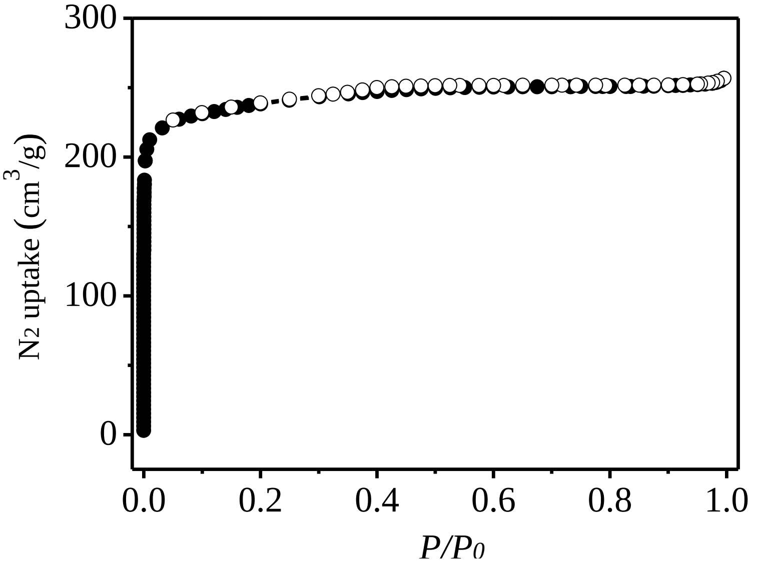

Supplementary Figure 15 | N_2 sorption isotherms of ZIF-8-VF after aging at 100% relative humidity and 45°C under CO_2 for 720 h. The well retained BET surface area and pore volume indicate that ZIF-8-VF sample is highly stable for long-term aging in a humid CO_2 environment.

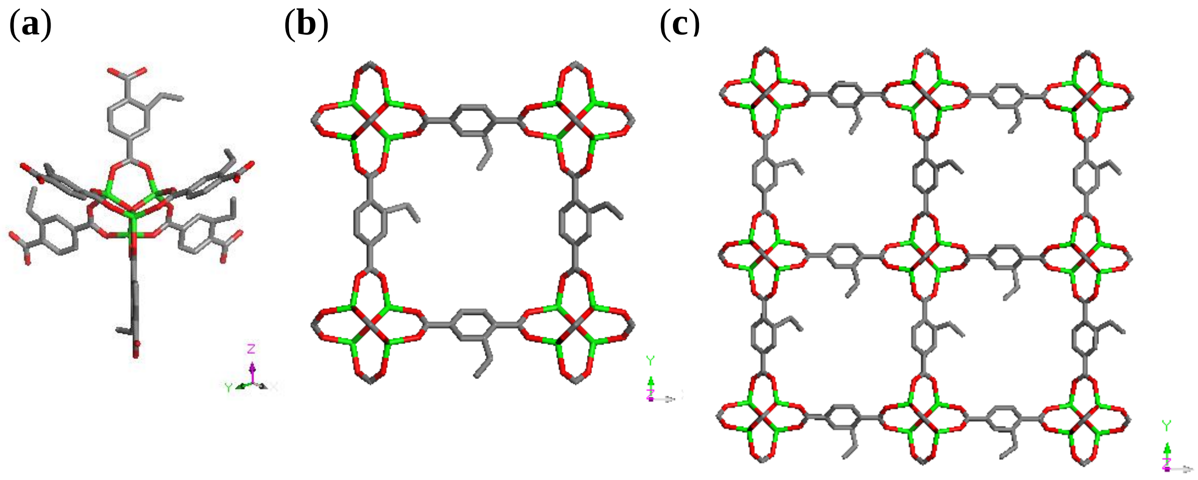

Supplementary Figure 16 | Representations of the crystal structure of MOF-5-V.

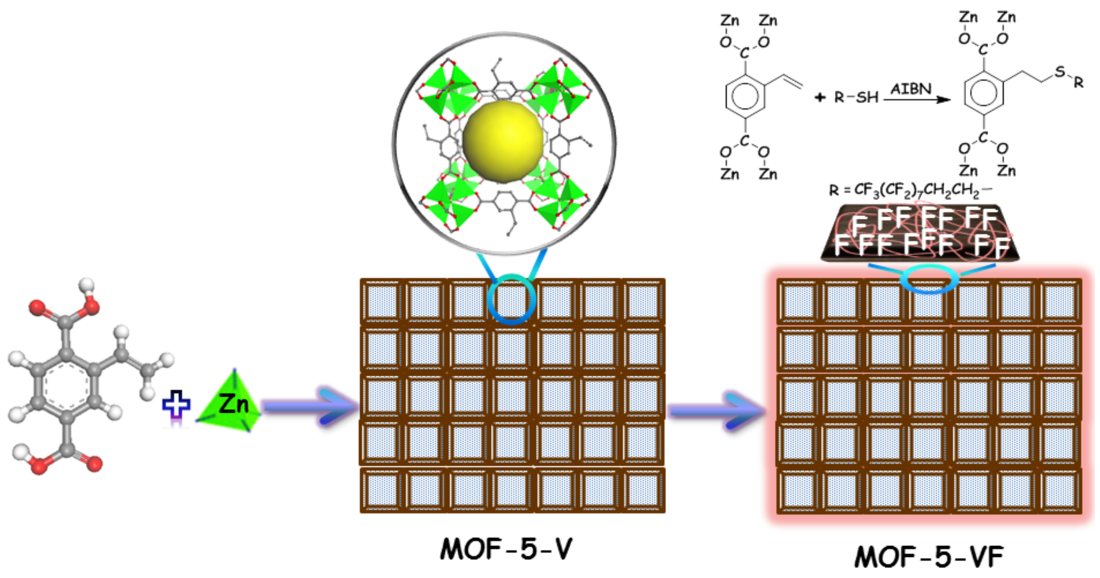

Supplementary Figure 17 | Synthetic route to amphiphobic MOF-5-V (MOF-5-VF). Illustration of creating amphiphobic surface via grafting perfluoroalkyl groups on the surface of the MOF-5-V crystal.

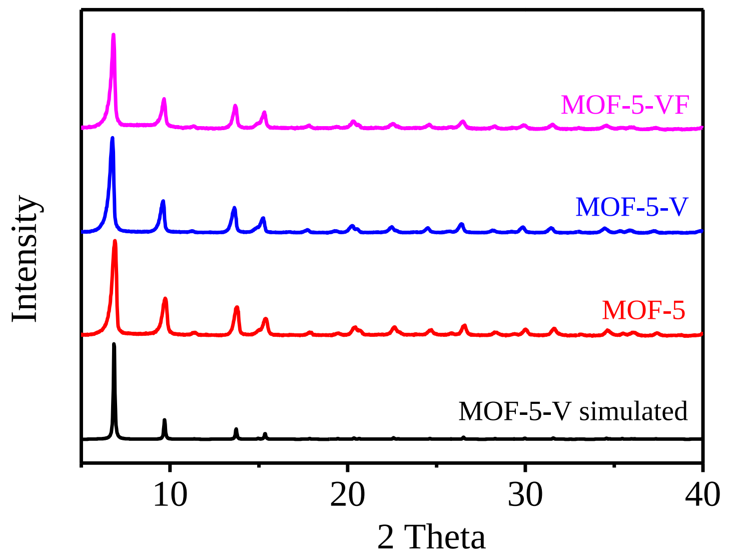

Supplementary Figure 18 | PXRD patterns. These PXRD patterns indicate that MOF-5-V has the same topology structure as MOF-5. After post synthetic modification, the structure of MOF-5-VF is retained, and the high crystallinity of the parent framework is also well maintained.

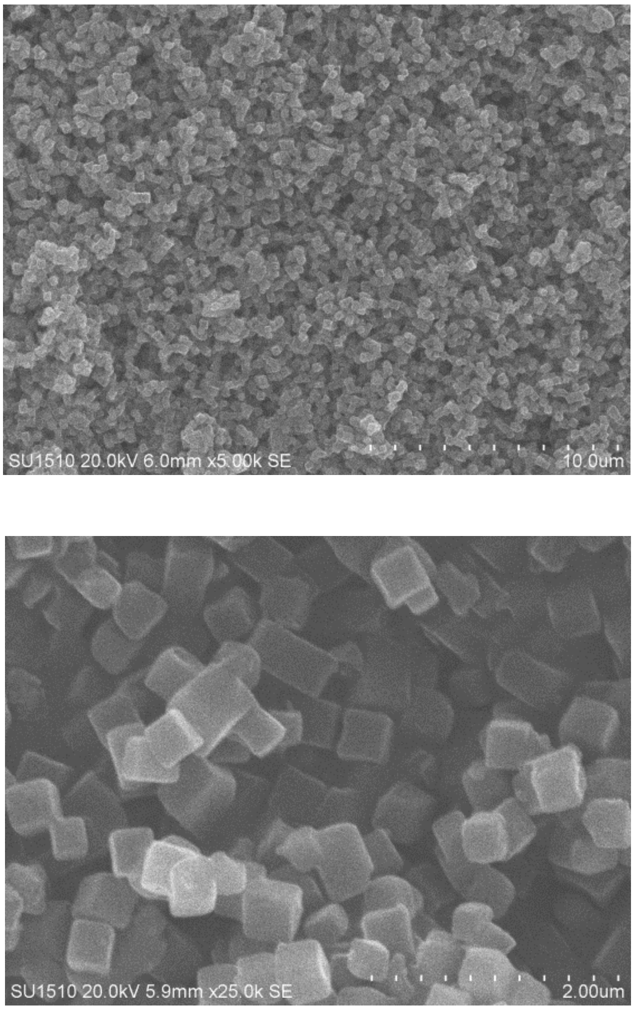

Supplementary Figure 19 | SEM images of MOF-5-V sample.

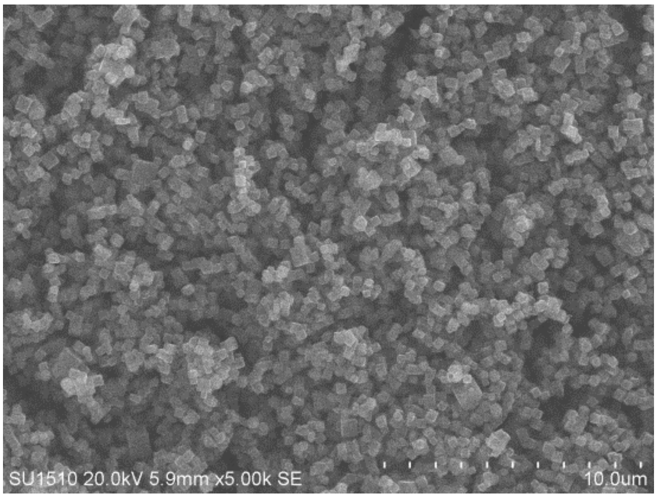

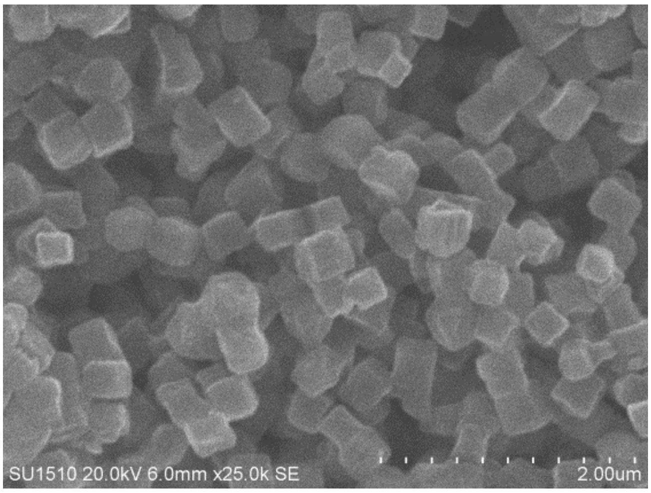

Supplementary Figure 20 | SEM images of MOF-5-VF sample. These pictures indicate that the crystal morphology is well retained after the introduction of perfluoroalkyl groups.

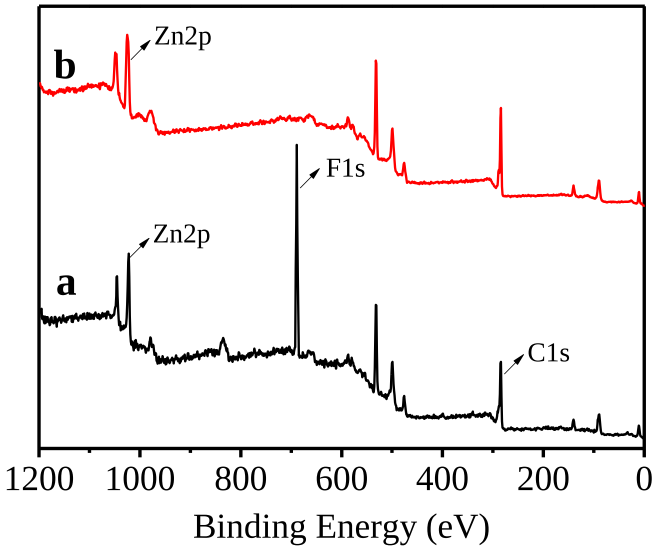

Supplementary Figure 21 | XPS spectra of MOF-5-VF sample. (**a**) before and (**b**) after surface Ar^+ ions etching. The strong signal of F species in the as-synthesized MOF-5-VF (around 23 atom%) sample can hardly be detected after surface Ar^+ ions etching, suggesting that 1H,1H,2H,2H-perfluorodecanethiol molecules are also mainly attached to the vinyl groups on the surface of the MOF-5-V crystals.

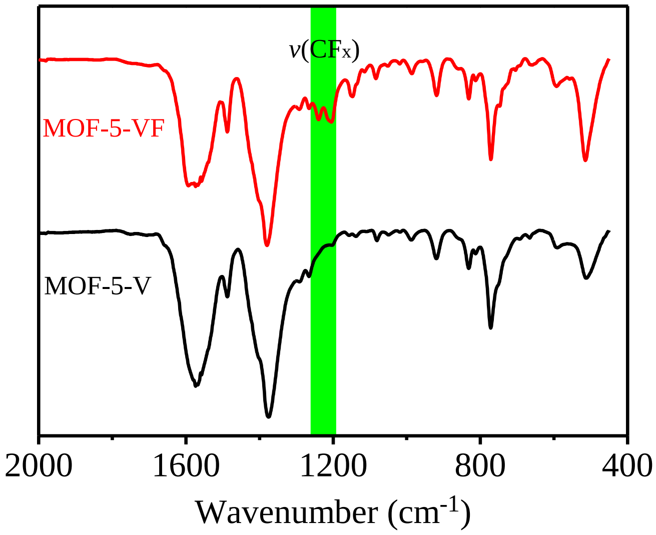

Supplementary Figure 22 | FT-IR spectra of MOF-5-V and MOF-5-VF. The appearance of characteristic bands of C-F at 1241 and 1211 cm^{-1} in MOF-5-VF indicates the successful grafting of perfluoroalkyl groups.[1]

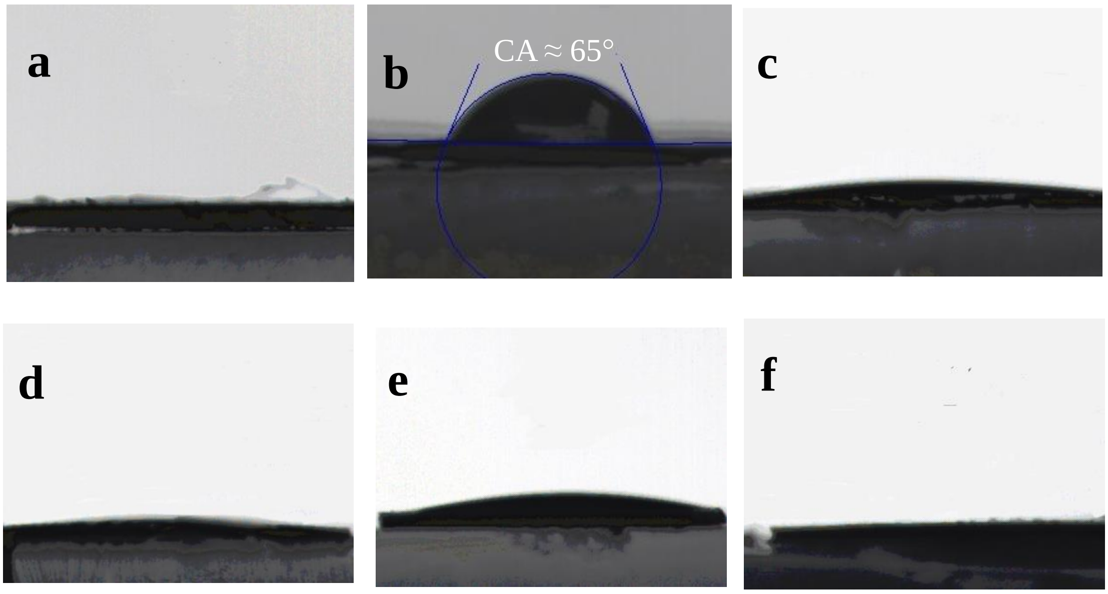

Supplementary Figure 23 | Contact angles of various compounds on the pressed pellet of MOF-5-V sample. (**a**) water, (**b**) glycerol, (**c**) 2-hydroxybenzaldehyde, (**d**) benzonitrile, (**e**) chlorobenzene, and (**f**) dodecane.

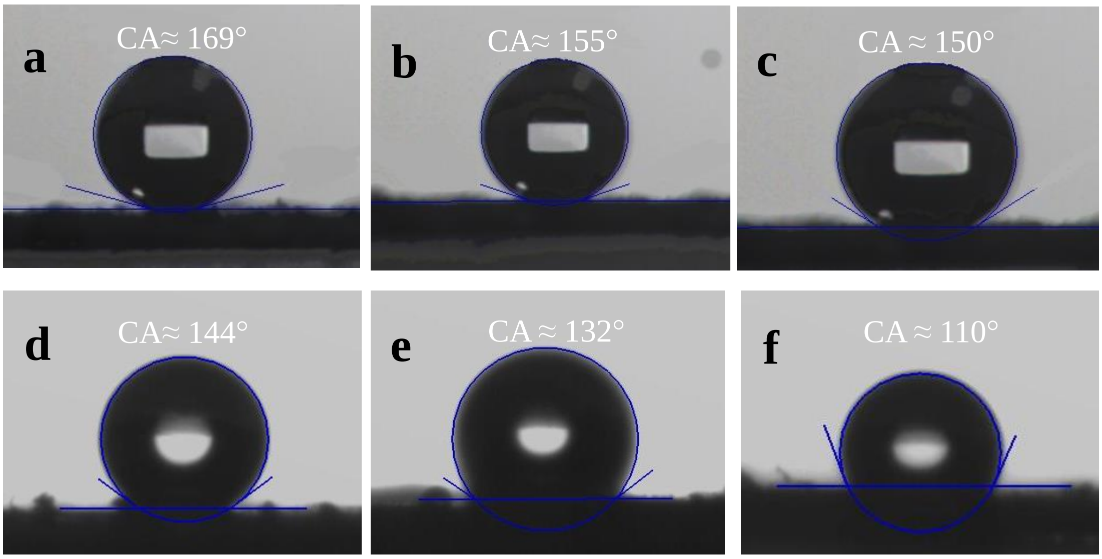

Supplementary Figure 24 | Contact angles of various compounds on the pressed pellet made of MOF-5-VF sample. (**a**) water, (**b**) glycerol, (**c**) 2-hydroxybenzaldehyde, (**d**) benzonitrile, (**e**) chlorobenzene, and (**f**) dodecane.

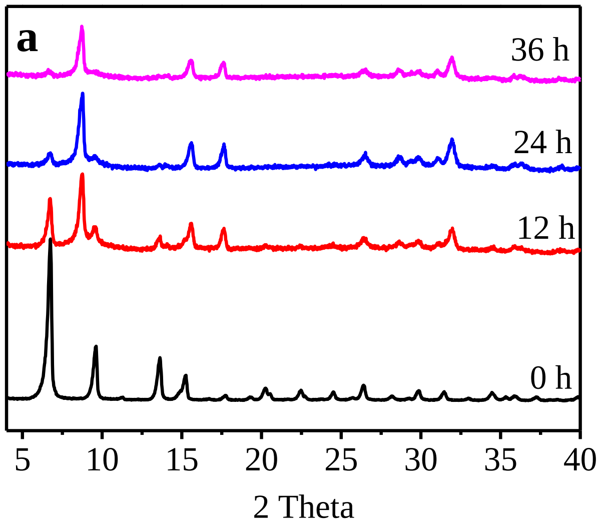

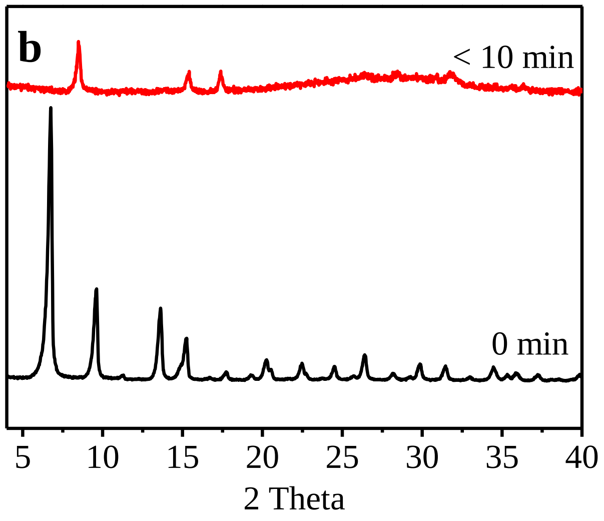

Supplementary Figure 25 | PXRD patterns of MOF-5 exposure to (a) 60% relative humidity, and (b) water for different time. These results indicate that MOF-5 is moisture sensitive.

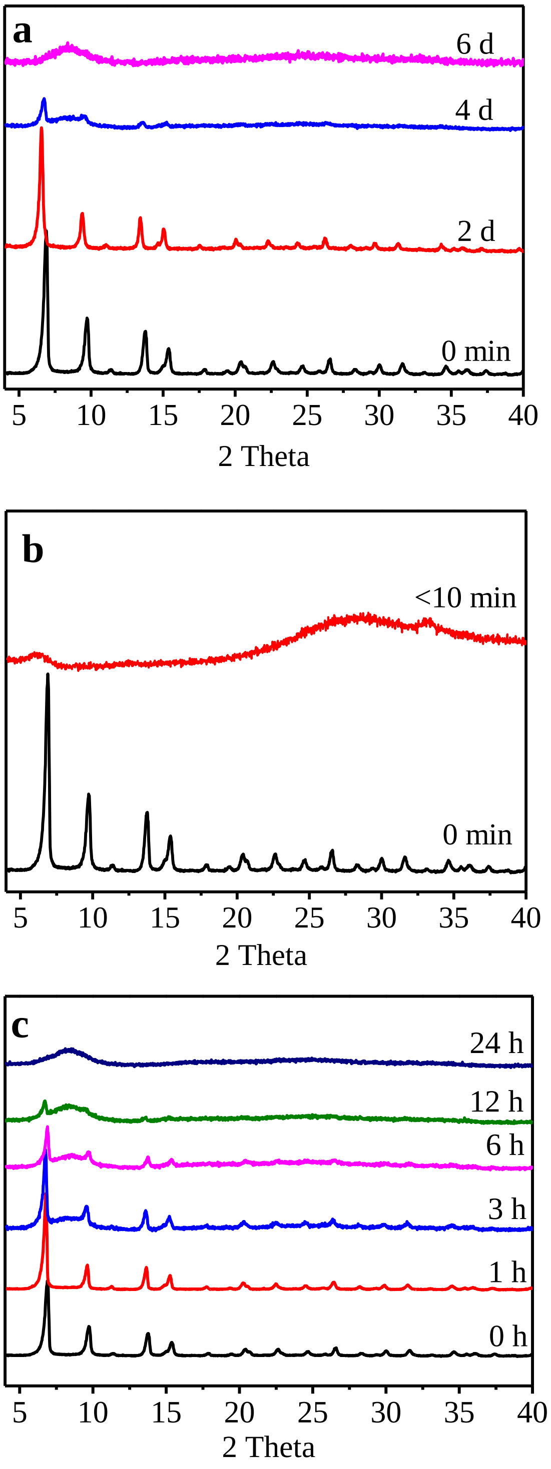

Supplementary Figure 26 | PXRD patterns of MOF-5-V exposure to various environments for different time. (**a**) 60% relative humidity, (**b**) water, and (**c**) 100% relative humidity and 45 °C under CO_2. These results indicate that MOF-5-V is moisture sensitive.

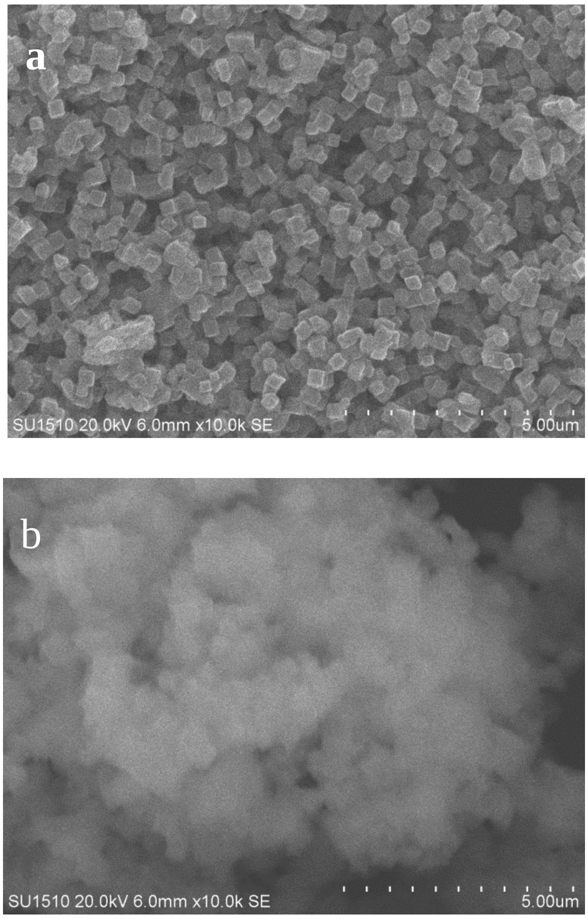

Supplementary Figure 27 | SEM images of MOF-5-V. (**a**) before and (**b**) after aging under 1 atm of water saturated CO_2 at 45 °C for 24 h.

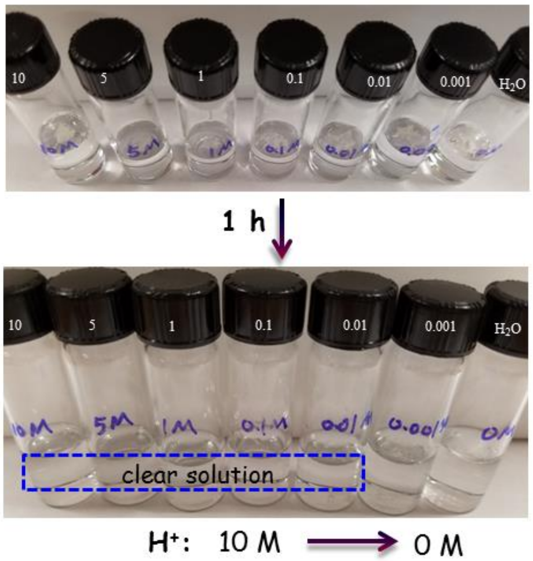

Supplementary Figure 28 | Stability tests of ZIF-8-V in acid solutions. Photos of ZIF-8-V crystals in various H_2SO_4 aqueous solutions with H^+ concentration from 10 M to 0 M. The ZIF-8-V crystals were digested within 1 h in the acid solutions with H^+ concentration higher than 0.01 M. PXRD studies indicate that ZIF-8-V can survive in acid solutions with H^+ concentration lower than 0.001 M for at least 7 days.

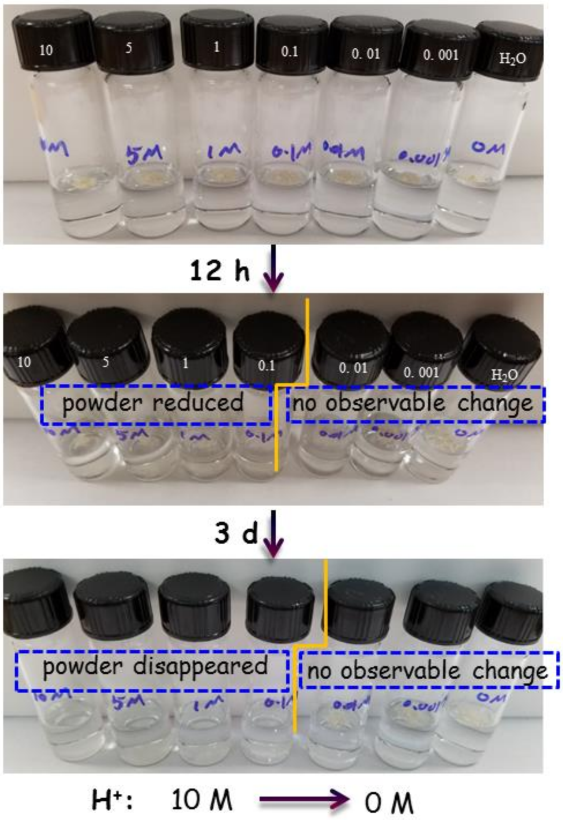

Supplementary Figure 29 | Stability tests of ZIF-8-VF in acid solutions. Photos of ZIF-8-VF crystals in various H_2SO_4 aqueous solutions with H^+ concentration from 10 M to 0 M. The ZIF-8-VF crystals float on all of the testing solutions, but observable digestion of the sample occurs under the acid conditions with the H^+ concentration higher than 0.1 M. No significant change was observed for the samples in the H^+ concentrations lower than 0.01 M. To make sure there is sufficient acid solution, a large amount of 0.01 M acid solution was used (3 mg of ZIF-8-VF crystals *vs* 50 mL of acid solution). After 7 days, the ZIF-8-VF crystals still floated on the solution and did not lead to observable structural change, which thus indicates that ZIF-8-VF can tolorate the H_2SO_4 solutions with H^+ concentration lower than 0.01 M. These results suggest that ZIF-8-VF is more stable than ZIF-8-V in acid solutions.

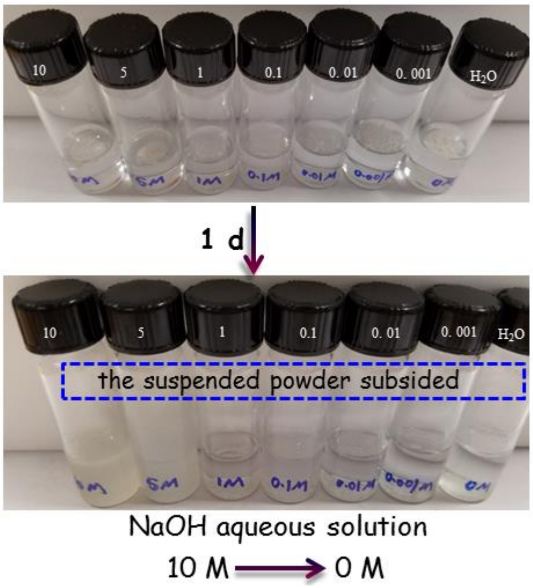

Supplementary Figure 30 | Stability tests of ZIF-8-V in basic solutions. Photos of ZIF-8-V in various NaOH aqueous solutions with OH^- concentration from 10 M to 0 M. The suspended ZIF-8-V crystals subsided after 1 day for all the solutions tested. PXRD results revealed that samples immersed in those solutions did not experience any significant change after 7 days, thus suggesting the retention of structural integrity.

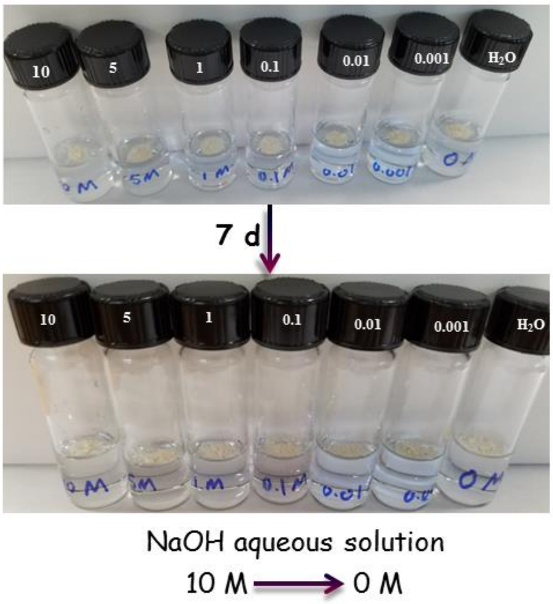

Supplementary Figure 31 | Stability tests of ZIF-8-VF in basic solutions. Photos of ZIF-8-VF crystals in various NaOH aqueous solutions with OH^- concentration from 10 M to 0 M. The ZIF-8-VF crystals float on all of the testing solutions and continue floating after 7 days. PXRD studies revealed no significant changes in the diffraction patterns for all samples after 7 days, thus indicative of no phase transition or framework collapse for ZIF-8-VF even in 10 M NaOH.

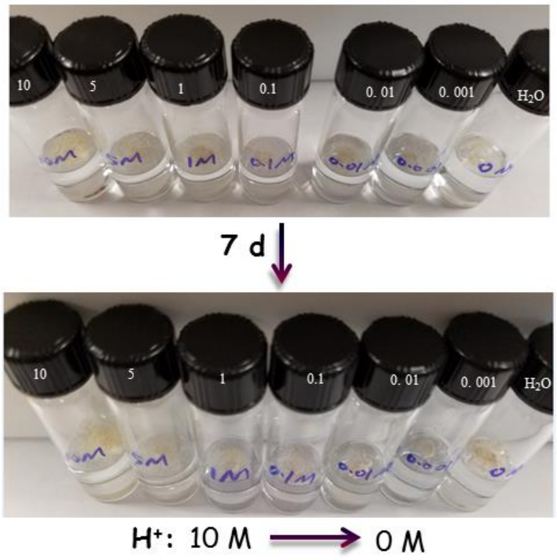

Supplementary Figure 32 | Stability tests of MOF-5-VF in acid solutions. Photos of MOF-5-VF crystals in various H_2SO_4 aqueous solutions with H^+ concentration from 10 M to 0 M. The MOF-5-VF crystals float on all of the testing solutions and continue floating for at least 7 days. PXRD patterns of all samples after 7 day's treatment are well consistent with the original ones, thus indicative of no phase transition or framework collapse for MOF-5-VF even in 10 M H_2SO_4. In sharp contrast, exposure to water led to complete degradation of the MOF-5V framework within 10 minutes, as evidenced by the rapidly vanishing PXRD peaks.

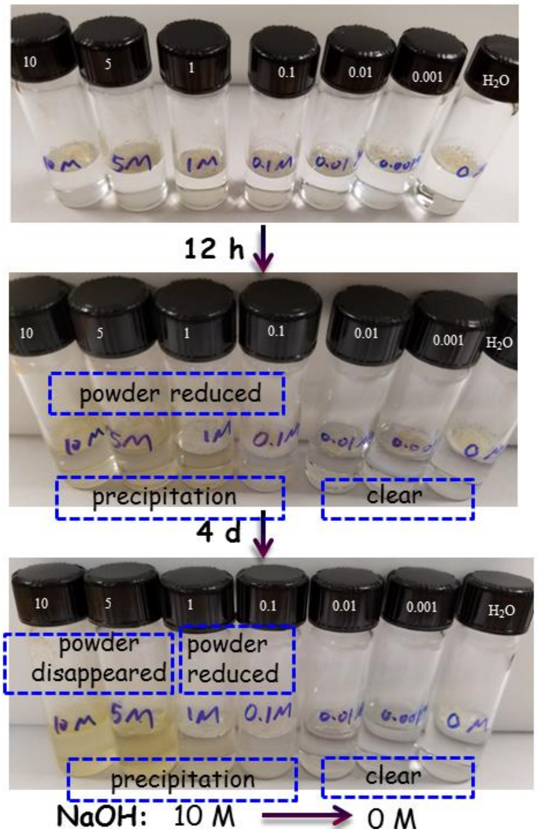

Supplementary Figure 33 | Stability tests of MOF-5-VF in basic solutions. Photos of MOF-5-VF crystals in various NaOH aqueous solutions with OH^- concentration from 10 M to 0 M. The MOF-5-VF crystals float on all of the testing solutions, but observable precipitation formed for those in the OH^- concentration higher than 0.1 M. PXRD results reveal that the formed precipitations are amorphous. With respect to the samples in the OH^- concentrations lower than 0.01 M, no observable change occurs and their PXRD patterns are well consistent with the original ones, which indicate that MOF-5-VF can tolerate the basic solutions with OH^- concentrations lower than 0.01 M.

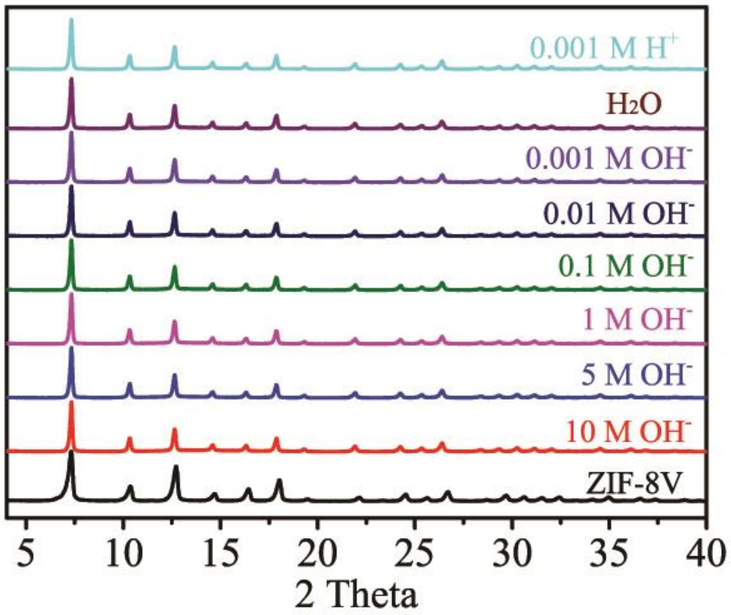

Supplementary Figure 34 | PXRD patterns of ZIF-8-V after treatments in acid and basic solutions with different concentrations for 7 days.

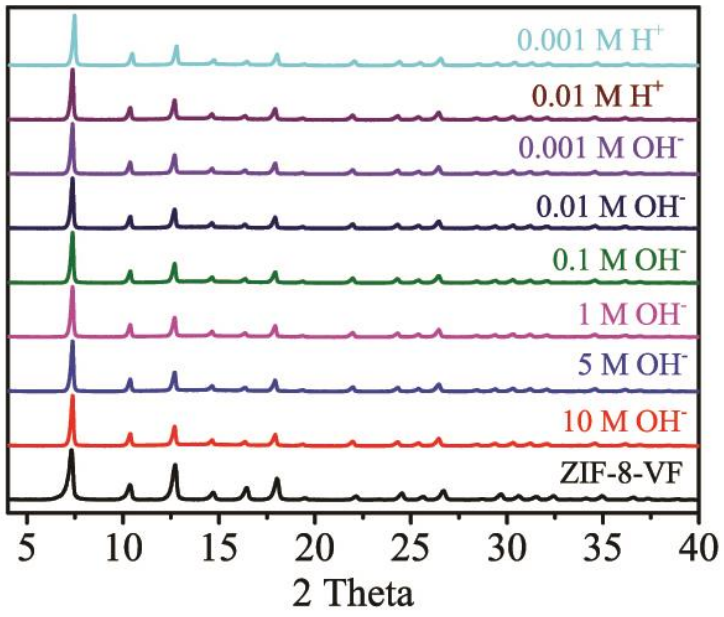

Supplementary Figure 35 | PXRD patterns of ZIF-8-VF after treatments in acid and basic solutions with different concentrations for 7 days.

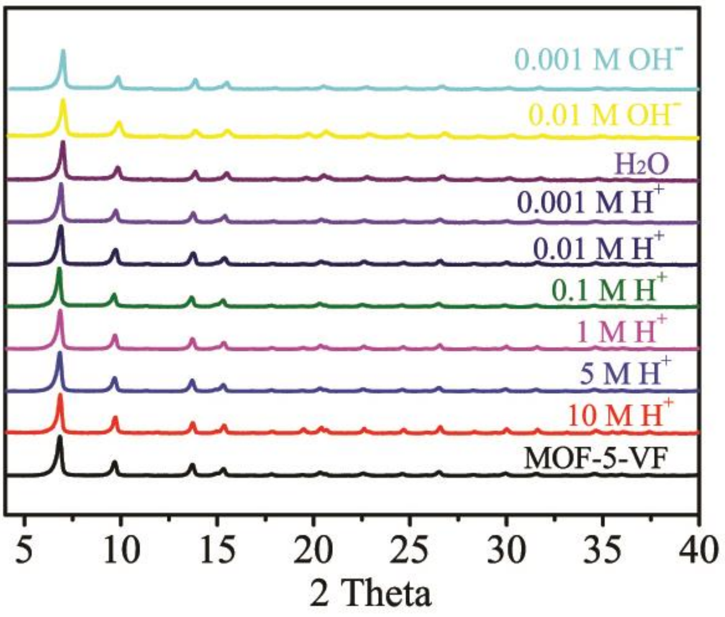

Supplementary Figure 36 | PXRD patterns of MOF-5-VF after treatments in acid and basic solutions with different concentrations for 7 days.

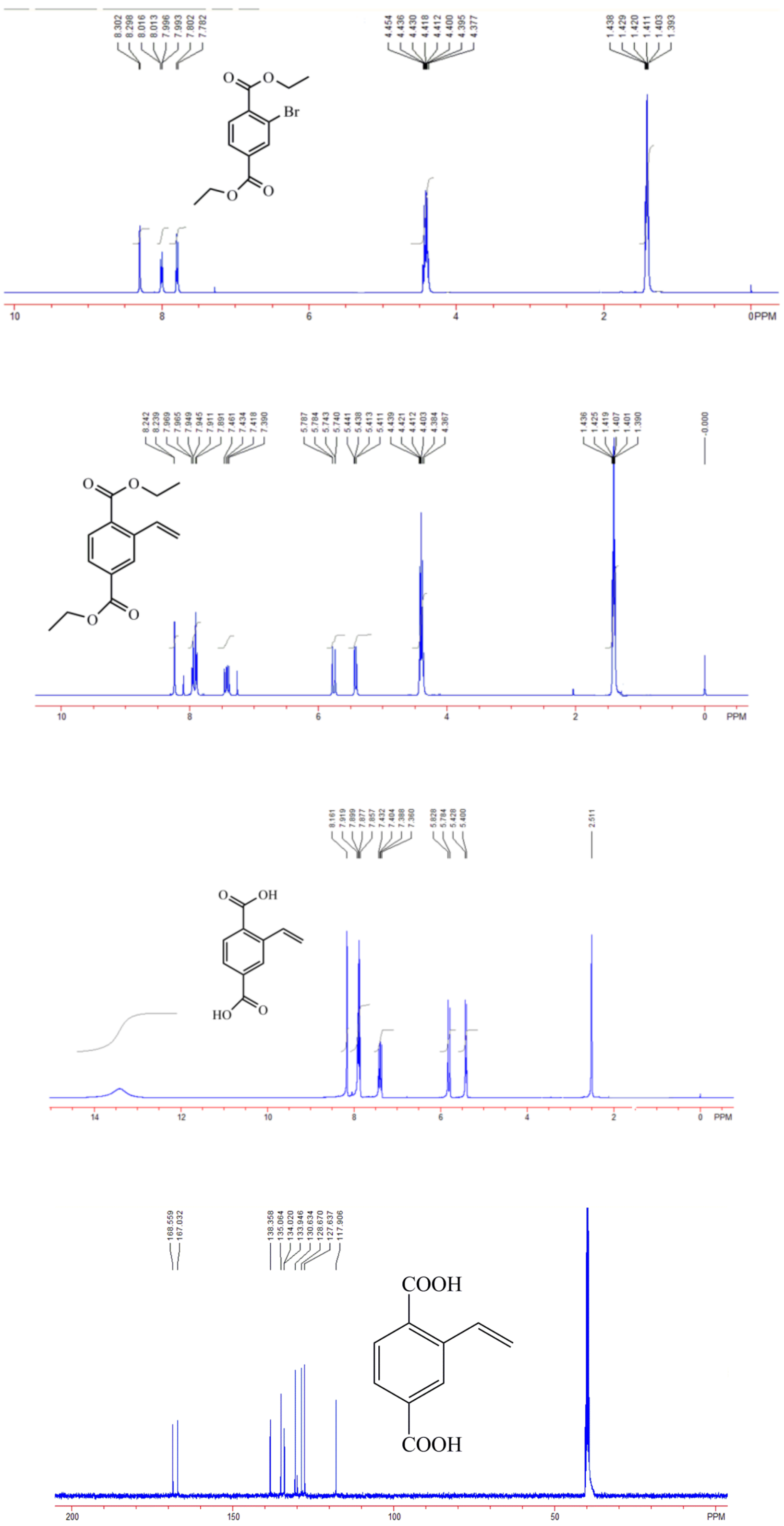

Supplementary Figure 37 | Liquid NMR spectra of various compounds.

Supplementary Tables

Supplementary Table 1 Crystal data and structure refinement for ZIF−8−V.	
Identification code	**ZIF-8-V**
Empirical formula	$C_{10}H_{10}N_4O_{2.38}Zn$
Formula weight	289.59
Temperature/K	100.0
Crystal system	cubic
Space group	I-43m
a/Å	17.1473(6)
b/Å	17.1473(6)
c/Å	17.1473(6)
α/°	90
β/°	90
γ/°	90
Volume/Å^3	5041.8(5)
Z	12
ρ_{calc}g/cm^3	1.145
μ/mm^{-1}	2.049
F(000)	1764.0
Crystal size/mm^3	0.06 × 0.06 × 0.06
Radiation	CuKα (λ = 1.54178)
2Θ range for data collection/°	10.318 to 117.6
Index ranges	-19 ≤ h ≤ 18, -17 ≤ k ≤ 17, -18 ≤ l ≤ 19
Reflections collected	11102
Independent reflections	711 [R_{int} = 0.0778, R_{sigma} = 0.0250]
Data/restraints/parameters	711/1/71
Goodness-of-fit on F^2	1.143
Final R indexes [I>=2σ (I)]	R_1 = 0.0586, wR_2 = 0.1777
Final R indexes [all data]	R_1 = 0.0828, wR_2 = 0.2128
Largest diff. peak/hole / e Å^{-3}	0.55/-0.55
Flack parameter	0.02(3)

**Supplementary Table 2 Crystal data and structure refinement for MOF−5−V.**	
Identification code	**MOF-5-V**
Empirical formula	$C_{24}H_9O_{13}Zn_4$
Formula weight	766.79
Temperature/K	100(2)
Crystal system	cubic
Space group	Fm-3m
a/Å	25.795(7)
b/Å	25.795(7)
c/Å	25.795(7)
α/°	90
β/°	90
γ/°	90
Volume/Å^3	17164(15)
Z	8
ρ_{calc}g/cm^3	0.593
μ/mm^{-1}	0.237
F(000)	3016.0
Crystal size/mm^3	0.04 × 0.04 × 0.04
Radiation	synchrotron (λ = 0.41328)
2Θ range for data collection/°	3.044 to 26.522
Index ranges	-28 ≤ h ≤ 22, -25 ≤ k ≤ 16, -28 ≤ l ≤ 28
Reflections collected	7721
Independent reflections	671 [R_{int} = 0.1641, R_{sigma} = 0.0676]
Data/restraints/parameters	671/8/36
Goodness-of-fit on F^2	1.041
Final R indexes [I>=2σ (I)]	R_1 = 0.0716, wR_2 = 0.2103
Final R indexes [all data]	R_1 = 0.1150, wR_2 = 0.2477
Largest diff. peak/hole / e Å^{-3}	0.35/-0.32

Supplementary Methods

Synthesis of 2-vinyl-imidazole.

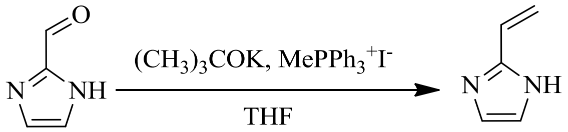

Methyltriphenylphosphonium iodide (42.0 g, 104 mmol, 2.0 equiv) was added to a suspension of $(CH_3)_3COK$ (12.2 g, 109 mmol, 2.1 equiv) in THF (150 mL) at room temperature under N_2 atmosphere. After 0.5 h, imidazole-2-carboxaldehyde (5.0 g, 52 mmol, 1.0 equiv) was added and the mixture was stirred at room temperature for another 24 h. After the reaction was quenched by the addition of NH_4Cl solution, the mixture was extracted with an excess of ethyl acetate, and then the combined organic phase was washed with brine and dried over $MgSO_4$. The residue was purified by flash column chromatography on silica gel to afford 2-vinyl-imidazole (4.2 g, 86%) as light yellow solid. ^{1}H NMR (400 MHz, $CDCl_3$, 298K, TMS): δ 11.75 (s, 1H), 7.08 (s, 2H), 6.62-6.70 (m, 1H), 5.93 (d, 1H, *J*=18 Hz), 5.33 (d, 1H, *J*=11.6 Hz) ppm. ^{13}C NMR (100 MHz, $CDCl_3$) δ 116.41, 122.62, 125.79, 146.36 ppm.

Synthesis of 2-vinylterephthalic acid.

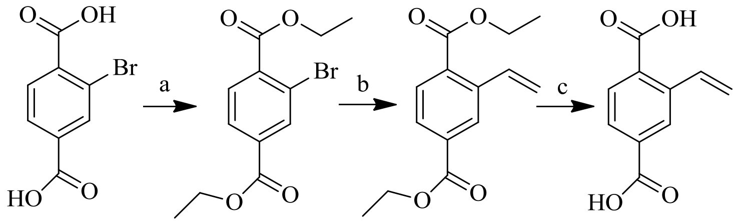

(1) (2) (3)

Reagents: (a) CH_3CH_2OH, H_2SO_4, 80 °C; (b) vinyltributyltin, $Pd(PPh_3)_4$, toluene, 100 °C; (c) $Li(OH)\cdot H_2O$, MeOH, H_2O, 60 °C.

diethyl 2-bromoterephthalate (1). A mixture of 2-bromoterephthalic acid (5 g, 20.5 mmol), concentrated H_2SO_4 (4.0 mL) and EtOH (100 mL) was refluxed for 12 h under N_2 atmosphere. The mixture was extracted with an excess of Et_2O, and then the combined organic phase was washed with $NaHCO_3$ aqueous solution, brine and dried over $MgSO_4$. The residue was purified by flash column chromatography on silica gel to afford diethyl 2-bromoterephthalate (5.62 g, 91%) as transparent liquid. ^{1}H NMR (400 MHz, $CDCl_3$, 298K, TMS): δ 8.3 (d, 1H, *J*=1.6 Hz), 7.99-8.02 (m, 1H), 7.79 (d, 1H, *J*=1.6 Hz), 4.38-4.45 (m, 4H), 5.33 (d, 1H, *J*=11.6Hz) ppm.

diethyl 2-vinylterephthalate (2). **1** (5.0 g, 16.7 mmol), vinyltributyltin (6.34 g, 20.0 mmol) and $Pd(PPh_3)_4$ (0.97 g, 0.83 mmol) were dissolved in dry toluene and the resulting mixture was refluxed at 100 °C under N_2 atmosphere for 24 h. The residue was purified by flash column chromatography on silica gel to the title compound as light yellow liquid (3.51 g, 85%).^{1}H NMR (400 MHz, $CDCl_3$, 298K, TMS): δ 8.24 (d, 1H, *J*=1.2 Hz), 7.89-7.97 (m, 2H), 7.39-7.46 (m, 1H), 5.74-5.79 (m, 1H), 5.41-5.44 (m, 1H), 4.37-4.44 (m, 4H), 1.39-1.44 (m, 6H) ppm.

2-vinylterephthalic acid (3). $LiOH·H_2O$ (37.8 g, 900 mmol) was added to a solution of **2** (2.00 g, 8.0 mmol) in MeOH-water (2:1; 80 mL). After the reaction mixture was stirred at 60 °C for 24 h, the solution was acidified with HCl (2.0 mol/L). The residual was extracted with an excess of EtOAc and the combined organic phase was washed with brine and dried over $MgSO_4$, filtered and concentrated under vacuum to yield **3** as a white solid (1.55g, quantitative yield). ^{1}H NMR (400 MHz, $CDCl_3$, 298K, TMS):

δ 13.5 (br, 1H), 8.16 (s, 1H), 7.86-7.92 (m, 2H), 7.36-7.43 (m, 1H), 5.81 (d, 1H, *J*=17.6 Hz), 5.41 (d, 1H, *J*=11.2 Hz) ppm. ^{13}C NMR (100 MHz, $CDCl_3$) δ 117.91, 127.64, 128.67, 130. 63, 133.95, 134.02, 135.06, 138.36, 167.03, 168.56 ppm.

Synthesis of single crystal MOF-5-V. Single crystal of MOF-5-V was obtained by heating a solution of $Zn(NO_3)_2 \cdot 6H_2O$ (0.3 mmol) and 2-vinylterephthalic acid (0.1 mmol) in the mixture of DMF (1 mL) and methanol (1 mL) for 12 h at 85 °C.

Synthesis of powder MOF-5-V crystal. Zinc acetate dihydrate (0.219 g, 1.0 mmol) diethylforamide (5.0 mL) solution was slowly added into the 2-vinylterephthalic acid (0.075 g, 0.39 mmol) diethylforamide solution (5.0 mL) under magnetic stirring at room temperature for 12 h. The crystalline powder was obtained by centrifugation, washing with DMF and $CHCl_3$ for several times, and activating with $CHCl_3$ (3×25 mL) for three-days before being dried under vacuum at room temperature. Elemental Analysis (activated sample): Calculated for $Zn_4O(C_{10}H_6O_4)_3$ (%): C, 42.49; H, 2.14. Found (%): C, 40.32; H, 2.25.

Synthetic procedure for ZIF-8 and MOF-5. ZIF-8 and MOF-5 were synthesized according to the previous reported procedures.[2,3]

Covalent post-synthetic modification of MOF-5-V. 1H,1H,2H,2H-perfluorodecanethiol coated MOF-5-V was operated according to the similar procedure to that of ZIF-8-VF. Elemental Analysis (activated sample): Found (%): C, 43.51; H, 1.96.

Digestion of ZIF-8-V and ZIF-8-VF: Approximately 10 mg of ZIF-8-V and ZIF-8-VF were digested by sonication in 1.0 mL of d_6-DMSO and 100 µL of 20% DCl in D_2O solution. Upon complete dissolution of the crystals, this solution was used for ^{1}H NMR analysis.

Single-Crystal X-ray diffraction studies: The X-ray diffraction data for ***ZIF-8-V*** were collected using Bruker D8 Venture PHOTON 100 CMOS system equipped with a Cu K_α INCOATEC ImuS micro-focus source (λ = 1.54178 Å). The X-ray diffraction data for ***MOF-5-V*** were collected using synchrotron radiation (λ = 0.41328 Å) at Advanced Photon Source Beamline 15-ID-B of ChemMatCARS in Argonne National Lab, Argonne, IL. Indexing was performed using *APEX2*[4] (Difference Vectors method). Data integration and reduction were performed using SaintPlus 6.01[5]. Absorption correction was performed by multi-scan method implemented in SADABS[6]. Space groups were determined using XPREP implemented in APEX2[4]. Structures were solved using SHELXS-97 (direct methods) and was refined using SHELXL-2015[10] (full-matrix least-squares on F^2) through OLEX2 interface program[11].

ZIF-8-V: All framework atoms were refined anisotropically. Hydrogen atoms were placed in geometrically calculated positions and were included in the refinement process using riding model. Disordered content of structural pores have been modeled as O atoms. The length of C7-C5 bond has been restrained using DFIX. No ADP restraints have been used. Crystal data and refinement conditions are shown in Supplementary Table 1.

MOF-5-V: The cluster and the ligand are disordered in the structure. The atoms of the major part of disorder were refined anisotropically. The minor part of disorder was refined isotropically and using restraints. The vinyl group has not been located due to disorder over approximately eight positions. The content of structural voids was not located due to the disorder. Crystal data and refinement conditions are shown in Supplementary Table 2.

Supplementary References

1. Qian, C.; Sun, W.; Wang, L.; Chen, C.; Liao, K.; Wang, W.; Jia, J.; Hatton, B.; Casillas, G.; Kurylowicz, M.; Yip, C. M.; Mastronardi, M. L. & Ozin, G. A. Non-wettable, oxidation-stable, brightly, luminescent, perfluorodecyl-capped silicon nanocrystal film. *J. Am. Chem. Soc.* **136**, 15849-15852 (2014).
2. Morris, W.; Doonan, C. J.; Furukawa, H.; Banerjee, R.; Yaghi, O. M. *J. Am. Chem. Soc.* **130**, 12626-12627 (2008).
3. Tranchemontagne, D. J.; Hunt, J. R.; Yaghi, O. M. *Tetrahedron* **64**, 8553-8557 (2008).
4. Bruker (2014). *APEX2* (Version 2013.6-2). Bruker AXS Inc., Madison, Wisconsin, USA.
5. Bruker (2013). SAINT-V8.32A. Data Reduction Software.
6. Sheldrick, G. M. (1996). *SADABS. Program for Empirical Absorption*
7. *Correction.* University of Gottingen, Germany.
8. Farrugia L.J. Appl. Cryst. (1999). 32, 837±838
9. Sheldrick, G.M. (1997) SHELXL-97. Program for the Refinement of Crystal
10. Sheldrick, G.M. (1990) Acta Cryst. A46, 467-473
11. Sheldrick, G. M. (2008). Acta Cryst. A64, 112-122.
12. Dolomanov, O.V.; Bourhis, L.J.; Gildea, R.J.; Howard, J.A.K.; Puschmann, H., OLEX2: A complete structure solution, refinement and analysis program. *J. Appl. Cryst.* **42**, 339-341(2009).
